# Co-design for stroke intervention development: Results of a scoping review

**DOI:** 10.1371/journal.pone.0297162

**Published:** 2024-02-14

**Authors:** Hardeep Singh, Natasha Benn, Agnes Fung, Kristina M. Kokorelias, Julia Martyniuk, Michelle L. A. Nelson, Heather Colquhoun, Jill I. Cameron, Sarah Munce, Marianne Saragosa, Kian Godhwani, Aleena Khan, Paul Yejong Yoo, Kerry Kuluski

**Affiliations:** 1 Department of Occupational Science & Occupational Therapy, Temerty Faculty of Medicine, University of Toronto, Toronto, Ontario, Canada; 2 The KITE Research Institute, Toronto Rehabilitation Institute-University Health Network, Toronto, Canada; 3 Rehabilitation Sciences Institute, Temerty Faculty of Medicine, University of Toronto, Toronto, Canada; 4 Dalla Lana School of Public Health, University of Toronto, Toronto, Canada; 5 Department of Medicine, Geriatrics Division, Sinai Health System, University Health Network, Toronto, Canada; 6 Gerstein Science Information Centre, University of Toronto Libraries, University of Toronto, Toronto, Canada; 7 Institute for Health Policy, Management and Evaluation, Dalla Lana School of Public Health, University of Toronto, Toronto, Canada; 8 Lunenfeld-Tanenbaum Research Institute, Sinai Health System, Toronto, Canada; 9 Department of Psychology, University of Toronto Scarborough, Toronto, Canada; 10 Biological Sciences, University of Toronto, Toronto, Canada; 11 Division of Neurosciences and Mental Health, The Hospital for Sick Children Research Institute, Toronto, Canada; 12 Institute for Better Health, Trillium Health Partners, Toronto, Canada; Universita di Bologna, ITALY

## Abstract

**Background:**

Co-design methodology seeks to actively engage end-users in developing interventions. It is increasingly used to design stroke interventions; however, limited guidance exists, particularly with/for individuals with stroke who have diverse cognitive, physical and functional abilities. Thus, we describe 1) the extent of existing research that has used co-design for stroke intervention development and 2) how co-design has been used to develop stroke interventions among studies that explicitly used co-design, including the rationale, types of co-designed stroke interventions, participants involved, research methodologies/approaches, methods of incorporating end-users in the research, co-design limitations, challenges and potential strategies reported by researchers.

**Materials and methods:**

A scoping review informed by Joanna Briggs Institute and Arksey & O’Malley methodology was conducted by searching nine databases on December 21, 2022, to locate English-language literature that used co-design to develop a stroke intervention. Additional data sources were identified through a hand search. Data sources were de-duplicated, and two research team members reviewed their titles, abstracts and full text to ensure they met the inclusion criteria. Data relating to the research objectives were extracted, analyzed, and reported numerically and descriptively.

**Results:**

Data sources used co-design for stroke intervention development with (n = 89) and without (n = 139) explicitly using the term ‘co-design.’ Among studies explicitly using co-design, it was commonly used to understand end-user needs and generate new ideas. Many co-designed interventions were technology-based (65%), and 48% were for physical rehabilitation or activity-based. Co-design was commonly conducted with multiple participants (82%; e.g., individuals with stroke, family members/caregivers and clinicians) and used various methods to engage end-users, including focus groups and workshops. Limitations, challenges and potential strategies for recruitment, participant-engagement, contextual and logistical and ethics of co-designed interventions were described.

**Conclusions:**

Given the increasing popularity of co-design as a methodology for developing stroke interventions internationally, these findings can inform future co-designed studies.

## Introduction

Stroke interventions designed solely by researchers may not always meet the complex needs of the end-users [1] (e.g., individuals with stroke and stroke caregivers and/or those who work in the field, such as clinicians who deliver stroke care, stroke care management, administrators, stroke organizations [2, 3]). A study found that only three percent of studies compare the researchers’ priorities with the end-users [4], resulting in stroke interventions lacking the end-users’ pragmatic insights into design development [5]. This may be a contributing cause of 85 percent of medical interventions not being implemented into clinical practice due to asking the wrong questions or biases within the research [6]. Participatory approaches, such as co-design, have been utilized to address this disconnect by incorporating end-users into all aspects of intervention development [7, 8]. For this review, co-design is broadly defined as a research methodology that actively engages end-users in developing a stroke intervention [5, 8–10]. Co-design has been an increasingly used methodology to enable shared responsibility and power for improved intervention design [7, 8]. By engaging end-users as equal contributors in the design and development stages, co-design can create a better and cohesive fit between the intervention and the context where the interventions will be used, leading to enhanced acceptability, adoption, and sustainability of the intervention and, thus potentially, improved patient outcomes and quality of care [8, 11–13].

With co-design, however, researchers face challenges of meaningfully engaging end-users, managing power imbalances and the additional time and resources required [12, 14–16]. These barriers may challenge the application of co-design in stroke research. For instance, individuals with stroke can have diverse post-stroke impairments related to communication (e.g., aphasia), function (e.g., hemiparesis) and cognition (e.g., interpersonal skills) [17–20], which may limit their participation in co-design [9]. Researchers must also consider and account for end-users’ differing abilities and needs when developing stroke interventions for individuals with stroke [10].

Current reviews have produced valuable insights, including recommendations, for improving the conduct of research co-design [8, 21–23]. These recommendations include training end-users in research skills, having regular and clear communication between end-users and researchers, and having the flexibility within budgets to adjust co-design activities to participant needs with cost [8, 24]. Various co-design methods have been used within health research, creating a lack of clarity on how this co-creation methodology may be used [8]. Within stroke intervention development, a recent review by Dobe and colleagues discussed the wide variance in co-design applications in the field [10]. Based on their review of 14 articles that used co-creation for stroke rehabilitation intervention development, the authors identified a need for future research to create consistency within co-design applications by providing clarity and direction for people using this methodology [10]. Further guidance is needed in the literature to inform the effective use of co-design methodology for stroke intervention development [25] due to the wide variation within the applications of co-design (e.g., [26–29]) and limited guidance for stroke research on its use [9, 30].

Despite these challenges, co-design is seen as vital for enhancing the impact of interventions within stroke research [27, 31–35], and there is a growing interest in understanding how a co-design methodology can best be used to develop stroke interventions [10, 30]. Given the varying associated concepts used to describe/define co-design (e.g., co-production and co-creation) and ways that co-design has been used in stroke intervention development (e.g., [27, 31, 32, 35]), a scoping review guiding this methodology would benefit future stroke intervention development that is consistent with the needs of the end-users [9, 30]. Thus, this scoping review aims to address current knowledge gaps by describing:

**Objective 1**: the extent of existing research that has used co-design for stroke intervention development, including the proportion of this literature that aligned with co-design or associated methodology with and without explicitly using the term co-design;

**Objective 2**: how it has been used to develop stroke interventions among studies that explicitly used co-design, including the rationale for using co-design, types of co-designed stroke interventions, participants involved in co-design, research methodologies/approaches to co-designing stroke interventions, methods of incorporating end-users in the research, co-design limitations, challenges and potential strategies reported by researchers.

The findings of this review have the potential to inform co-design studies used in future stroke intervention development for enhanced acceptability, sustainability, and adoption.

## Materials and methods

This scoping review is guided by the Joanna Briggs Institute [36] and Arksey & O’Malley [37] [9]. The PRISMA Extension for Scoping Reviews (PRISMA-ScR) was followed to enhance reporting quality [38]. As per our protocol [9], in the current review, we sought to answer the following research questions: 1) What is the extent of stroke research that used co-design or associated methodology with and without explicitly using this term? 2) How has co-design methodology been used to develop stroke interventions, including the rationale for using co-design, types of co-designed stroke interventions, participants involved in co-design, research methodologies/approaches to co-designing stroke interventions, methods of incorporating end-users in the research, co-design limitations, challenges and potential strategies reported by researchers?

Relevant English-language data sources were identified using peer-reviewed database searches [39] run by JM (Librarian) on the following databases (search date: December 21, 2022): Medline, Embase, PsychInfo, Cumulative Index to Nursing and Allied Health Literature, Scopus, Global Index Medicus, Cochrane Reviews, Cochrane Protocols and Cochrane Trials (see S1 Appendix). The database searches aimed to capture data sources that met our inclusion criteria. Specifically, published and gray literature (i.e., conference abstracts and dissertations) were included if they used a methodology that aligned with our broad definition of co-design (i.e., research methodology used to actively engage end-users in the development of a stroke intervention) [5, 8, 9]. Since co-design may be used interchangeably with several concepts, such as co-creation and co-production [10, 25], data sources did not have to explicitly use the term ‘co-design’ to be included in this review. End-users were defined as individuals impacted by stroke, including adults ≥18 years of age who experienced a stroke, caregivers/family members for adults with stroke, funders, clinicians, and other relevant end-users who work in the field of stroke. As the focus of this review was stroke intervention development, we excluded data sources seeking to co-design assessments, questionnaires, or frameworks (i.e., those not designing a specific stroke intervention). We also excluded pharmaceutical or surgical stroke interventions. No restrictions were imposed on study design (e.g., mixed methods, qualitative, quantitative studies) and context (e.g., date or study setting); however, book chapters and other reviews were excluded. In addition to the database searches, a research team member (NB) examined the reference lists of 10 included data sources to reduce the risk of excluding relevant literature. Two research team members (HS and AF) also searched ResearchGate and Google Scholar [40] to locate full texts of included 42 conference abstracts. The search results were uploaded into Covidence for deduplication, screening, and data extraction.

Before the screening, all research team members pilot-tested the inclusion criteria using ten randomly selected data sources to achieve 80% agreement before independently screening titles, abstracts, and full texts. HS screened all data sources during the title, abstract, and full-text review stages to ensure the inclusion criteria were consistently applied. HS also resolved screening conflicts during biweekly team meetings with the other screeners.

The data extraction form was informed by the Guidance for Reporting Involvement of Patients and the Public (a reporting checklist to describe the patient and public involvement in research) [41] and pilot-tested on three data sources for further refinement by HS. For example, if information regarding the country in which the study was conducted was not reported/unclear, information was extracted regarding the country from the first author’s affiliation. A research team member extracted data from each included study, and a second research team member reviewed the data for quality assurance. The extracted data relating to Objective 1 were described numerically (e.g., year of publication) and descriptively (e.g., terms used to describe end-user involvement). Objective 2 focused on a subset of data sources (i.e., those that explicitly used co-design) because we aimed to conduct an in-depth analysis of co-design methodology for stroke intervention development. Objective 2 was addressed numerically (e.g., describing the frequency of the most common co-design methodologies and methods) and descriptively by applying descriptive qualitative content analysis with pre-developed codes informed by prior literature on co-design approaches in health research [8, 42] using Microsoft Excel and NVivo 12. For instance, we coded relevant data from the included sources within codes, such as recruitment, activities, facilitators, methods, and challenges/limitations (i.e., recruitment-related, participant-engagement, contextual, logistical and ethical). Our research team synthesized a list of ‘potential strategies to overcome limitations and challenges’ by organizing the co-design methods and strategies reported by authors of the included studies according to the identified challenges and limitations.

## Results

### Description of the extent of existing research that has used co-design methodology for stroke intervention development with and without explicitly using the term co-design (Objective 1)

**Fig 1:**
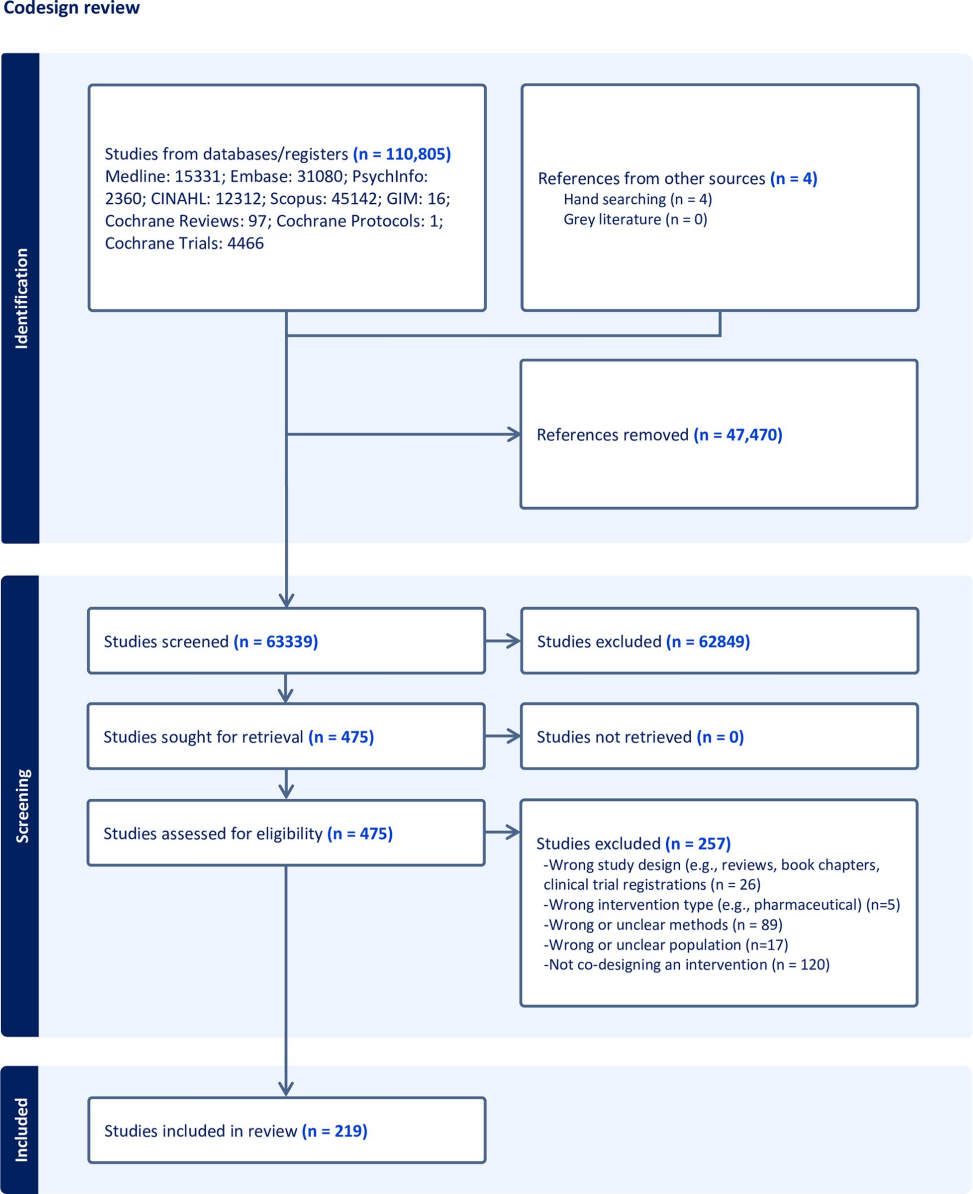
PRISMA flow diagram.

After deduplication in Covidence, 63,328 titles and abstracts were screened (see Fig 1 for the PRISMA flow diagram). A total of 219 data sources (i.e., full-length articles (n = 114/220, 52%), conference abstracts (n = 100/219, 46%), and dissertations (n = 5/219, 2%) met the inclusion criteria (see S1 Table). Data sources were published between 1996–2023: 1996–2005 (n = 1/219, >1%), 2006–2015 (n = 35/219, 16%), and 2016–2023 (n = 183/219, 84%).

Of the 219 data sources, 89 (41%) explicitly stated co-design. In comparison, the remaining data sources used a methodology that aligned with the definition of co-design [5, 8, 9] but did not explicitly use the term co-design. They used the following terms to describe the involvement of end-user participants in the design of interventions, with many using multiple terms: (co)create, (co)develop, (co)produce, collaborate, input/feedback/identify requirements, partner, engage, consult, inform, modify, refine, brainstorm, assist, establish, review, and partner, consult and engage.

### Description of data sources that explicitly used the term co-design

The following sections report the results of the data sources that explicitly used the term co-design. In total, 89 data sources (see Table 1), including 45 full-length articles (51%), 40 conference abstracts (45%), and four dissertations (4%) explicitly stated that they used the term co-design. These data sources were published between 2011–2023, with most published between 2021–2023 (n = 46/89, 52%) and 2016–2020 (n = 39/89, 44%), and a few between 2011–2015 (n = 4/89, 4%). Data sources were from the following countries: the UK (n = 36/89, 40%), Australia (n = 20/89, 22%), Sweden (n = 6/89, 7%), Canada (n = 4/89, 4%), US (n = 3/89, 3%), Spain (n = 2/89, 2%), Italy (n = 2/89, 2%), Ireland (n = 2/89, 2%), Scotland (n = 1/89, 1%), Singapore (n = 1/89, 1%), Portugal (n = 1/89, 1%), Netherland (n = 1/89, 1%), Iceland (n = 1/89, 1%), France (n = 1/89, 1%), Ethiopia (n = 1/89, 1%), China (n = 1/89, 1%), and the remaining five (6%) were conducted in multiple countries. Most data sources (n = 69/89, 78%) did not define co-design. The data sources that defined co-design indicated it is a participatory approach and methodology that seeks to engage various end-users actively and collaboratively in designing/re-designing interventions to create interventions that respond to their needs and improve user experiences [27, 29, 35, 43–57].

**Table 1:** Overview of 89 studies analyzed in this review that explicitly used the term co-design.

First author, Year	Summary of aims	What was co-designed
Ahmed, 2022 [58]	To create transformational change in a community stroke rehabilitation service	Stroke service-care portal, stroke journal, virtual prevention sessions, self-management support
Aljaroodi, 2017 [59]	To co-design a stroke rehabilitation mobile health artifact to enhance engagement in stroke rehabilitation	Stroke rehabilitation mobile health artifact
Alves, 2020 [60]	To design and evaluate a gaming approach for upper limb rehabilitation through observations, co-design workshops, interviews, and usability testing with health professionals	Upper-limb rehabilitation prototype
Anemaat, 2021 [43]	To use experience-based co-design to develop aphasia service elements and care pathways with people with aphasia, their families and clinicians	Aphasia service elements and care pathways
Armstrong, 2022 [61]	To report via a presentation the development and progress of support groups for Indigenous people with brain injury and their families to promote social and emotional well-being and health	Culturally secure support groups (yarning circles) for Aboriginal people with brain injury and family members for psycho-social support, education, practical problem-solving, yarning and socialization
Auger, 2022 [27]	To co-design a program for post-stroke sexual rehabilitation with people with stroke, partners, clinicians, managers and researchers	Sexual rehabilitation services program
Bagot, 2017 [62]	To describe the experiences of transitioning from a single-site pilot project to a regional telehealth service	Regional telehealth service (Victorian Stroke Telemedicine (VST) program)
Bajuaifer, 2021 [28]	To co-design a lower limb mirror therapy equipment and setup with individuals who have had a stroke and physiotherapists	Lower limb mirror stroke therapy equipment
Blanco, 2019 [63]	To design, develop, and assess a social-based solution for supporting patients with chronic disease	Social-based solution to support individuals with chronic diseases, including stroke (Micro ad hoc Health Social Networks)
Brandy, 2021 [64]	To co-develop a stroke self-management tool for the First Nations People of Australia	Culturally relevant stroke recovery self-management tool for First Nationals People of Australia (’Take Charge’ tool)
Brown, 2022 [65]	To develop a goal-setting resource for people with aphasia	Resource for goal setting and action planning for people with stroke aphasia (Access G-AP)
Calder, 2022 [44]	To co-design a prototype to guide/empower female partners involved in supporting males with stroke	Educational resource prototype
Cameron, 2020 [66]	To co-design an interdisciplinary team development program that improves collaboration and evidence-based service delivery related to promoting the engagement of people with stroke in meaningful self-care and functional activities	Interdisciplinary team program to facilitate evidence-based rehabilitation in an acute stroke setting
Carragher, 2018 [67]	To describe the utility of a co-designed communication app	App to support communication between clinicians and individuals with aphasia (Aphasia App)
Choo, 2020 [68]	To co-design stroke rehabilitation units to promote physical activity with end-users (e.g., individuals with stroke, clinicians, and carers)	Design of a stroke rehabilitation unit to promote physical activity
Clarke, 2019 [69]	To explore the use of experience-based co-design and accelerated experience-based co-design for developing interventions to increase activity opportunities for inpatients with stroke	Interventions to increase activity opportunities for inpatients with stroke
Clarke, 2021 [45]	To report the facilitators and barriers to using experience-based co-design and accelerated experience-based co-design to develop and implement a stroke intervention for use in acute stroke units	Interventions to increase activity opportunities for inpatients with stroke
Clatworthy, 2021 [70]	To co-design an intervention for visual field loss with individuals with stroke and clinicians	Intervention operationalizes best practice in OT for visual field loss after stroke (HABIT)
Cregg, 2021 [71]	To describe an adaptation to an online neurorehabilitation program	Cogs in motion: online cognitive rehab resources
Donetto, 2021 [29]	To describe an experience-based co-design project that intended to improve rates of patient activity within an acute stroke unit	Interventions to increase activity opportunities for inpatients with stroke
Drummond, 2020 [72]	To describe the co-design of a stroke fatigue management intervention	Intervention for post-stroke fatigue
Duval, 2023 [73]	To describe approaches to the design of play-based health intervention	Play-based health intervention
El-Helou, 2022 [46]	To identify the perspectives of people with post-stroke aphasia to inform the development of technology-based relaxation intervention	“Kalmer” relaxation intervention for people with aphasia
English, 2021 [74]	To evaluate a telehealth physical and dietary intervention	Telehealth physical activity and dietary (DIET) intervention
Fini, 2022 [75]	To co-design a physical activity intervention for individuals with stroke	Personalized physical activity intervention for people after stroke
Flynn, 2018 [76]	To develop a physical activity behavioural stroke intervention to be used by stroke rehabilitation teams	Sedentary behavioural intervention for use by stroke rehabilitation teams: Physical Activity Routines after Stroke (PARAS)
Fusari, 2020 [77]	To determine the feasibility of a co-designed digital upper limb stroke rehabilitation system (protocol)	Digital upper limb stroke rehabilitation system (OnTrack)
Gall, 2020 [78]	To co-design and pilot a stroke-adapted cardiac rehabilitation program	Adaptation of cardiac rehabilitation for people with stroke
Gombert, 2018 [47]	To use experience-based co-design to develop and implement service changes to increase social, cognitive and physical activities for individuals with stroke in acute stroke rehabilitation	Service changes to increase opportunities for patients to engage in social, cognitive and physical activities in inpatient stroke settings
Gregor, 2023 [48]	To develop a rhythm training program for people with stroke	Tool for Rhythm training for people with stroke
Gustafsson, 2022 [79]	To co-develop self-management support with individuals discharged from the hospital after a stroke	Self-management support across the care continuum
Harding, 2018 [80]	To describe the co-design and co-delivery of stroke information sessions	Stroke information sessions
Hopkins, 2018 [81]	To test a novel method of designing and developing stroke rehabilitation products with clinicians	Virtual reality using wearable headsets, mirror therapy and warble motion sensor technology
Hughes, 2019 [82]	To describe usability testing the acceptability of an upper extremity stroke telerehabilitation system	Stroke Rehabilitation mHealth Application
Jarvis, 2021 [83]	To test the virtual engagement rehabilitation assistant in an inpatient rehabilitation setting	Digital technology -Virtual Engagement Rehabilitation Assistant (VERA)
Jarvis, 2022 [84]	To evaluate the feasibility, usability, and acceptability of a co-designed virtual engagement rehabilitation assistant on a complex rehabilitation unit	Digital health technology in complex rehabilitation (VERA)
Jie, 2020 [85]	To describe the approach used to design a sensor feedback system to promote walking after stroke	A sensor feedback system to facilitate walking in people after stroke (Stappy)
Jones, 2018 [86]	To co-design changes to stroke units that will enhance the therapeutic environment and increase activity	Intervention to enhance physical activity in acute stroke units
Jones, 2019 [87]	To determine the feasibility and impact of an accelerated experience-based co-design approach to design an intervention to improve activity in stroke units	Intervention to enhance physical activity in acute stroke units
Jones, 2020 [50]	To describe the co-design of an intervention to enhance physical activity in acute stroke units and evaluate the feasibility of the co-design process and intervention impact	Intervention to enhance physical activity in acute stroke units
Jones, 2021 [49]	To evaluate the feasibility and impact of co-designing and implementing improvements in stroke units and compare the use of full and accelerated experience-based co-design approaches	Breakfast intervention three acute stroke units
Jones, 2021 [88]	To co-design and pilot test a breakfast group intervention and toolkit in three acute stroke units	Intervention to enhance physical activity in acute stroke units
Kelliher, 2019 [89]	To describe the co-design process used to create modular therapy objects and a rehabilitation protocol for upper extremity stroke rehabilitation	Modular Rehabilitation Objects for Interactive Therapy in the Home
Kilbride, 2018 [90]	To evaluate the safety, feasibility and acceptability of a co-designed upper extremity stroke rehabilitation gaming intervention	Home-based gaming exercise for the Upper limb post-stroke
Kilbride, 2019 [91]	To gather feedback from individuals with stroke on an upper limb rehabilitation prototype	Virtual hand and arm rehabilitation following stroke (Gameball)
Kingsley, 2011 [92]	To describe the co-design of solutions to create awareness of aphasia	Tools to co-create awareness about aphasia
Kjork, 2021 [93]	To evaluate a digital tool (the last step in their co-design)	Digital tool to support follow-up after stroke (Strokehealth)
Kjork, 2022 [51]	To describe the development of a digital previsit tool and explore perceptions of people with stroke	Pre-visit preparation digital tool (Strokehälsa [Strokehealth] version 1.0)
Langford, 2022 [94]	To describe the methodology used to co-design a digital therapy prototype	Digital therapy for people with aphasia (iReadMore)
Lee, 2022 [95]	To describe the iterative design of a physical stroke rehabilitation intervention	1) human-AI collaborative decision-making on rehabilitation assessment for therapists and (2) human-robot collaborative stroke rehabilitation therapy
Lievesley, 2022 [35]	To describe the co-design of a stroke behaviour change intervention for oral care	Oral-care intervention for people with stroke
Lindblom, 2021 [52]	To describe hospital-to-home stroke transition experiences and the co-design of a transition intervention	Stroke transition intervention
Lindblom, 2021 [96]	To describe user participation in the co-design of a stroke transition intervention	Stroke care transition intervention
Maddahi, 2021 [97]	To describe the design and development of a portable hand telerehabilitation platform for people with stroke and therapists’ perspectives on implementing it	Telerehabilitation platform for home-based personalized stroke rehabilitation
Magnusson, 2017 [98]	To describe the design of rehabilitation technology for people with stroke	Stroke rehabilitation technology (tangible interactive objects)
Magnusson, 2018 [99]	To report the development, initial testing and future development of an outdoor activity game for people with stroke	Step counting game for people with stroke
Markle-Reid, 2022 [100]	To explore the feasibility, usability, and benefits of a co-designed website to support stroke navigation for older adults with stroke	Care transition intervention (my stroke recovery journey website)
Masterson-Algar, 2020 [53]	To co-design and test a peer-led stroke coaching intervention to improve leisure and social participation after stroke	Peer-lead stroke coaching intervention
Mawson, 2014 [101]	To describe the user-centred, participatory approach to designing and evaluating a personalized stroke self-management system	Personalised self-management system for post-stroke rehabilitation (the Personalised Self-Managed Rehabilitation System (PSMrS))
McGowan, 2021 [102]	To describe the development of a stroke information website	Stroke informational website (EnableMe)
McGowan, 2022 [103]	To describe a project that co-designed and co-delivered online information for adults with stroke	Online stroke-related information (Stroke Foundation’s Young Stroke Project)
Nasr, 2016 [104]	To describe opportunities to co-design stroke technology with end-users	Robotic technology for home-based rehabilitation of the hand and wrist
Neves, 2021 [54]	To describe the co-design of a dietary intervention	Intervention to improve mealtime in stroke rehabilitation
Ng, 2019 [105]	To develop a transition process through co-design	Discharge tools: Patient-Oriented Discharge-Summary
Olafsdottir, 2020 [55]	To describe the development of a virtual intervention for home-based physical activity for people with stroke	Virtual home-based exercise for people with stroke (Activables)
O’Malley, 2022 [106]	To describe the Stroke Foundation’s Young Stroke Project (a co-designed project) that provides information to adults with stroke	Information for individuals with stroke
Ortiz-Fernandez, 2019 [107]	To co-design a stroke self-management app	“Decision SupporT and Self-Management System for StRoke SurvivoRs” (STARR) for self-management and reduction of recurrent stroke
Pogrebnoy, 2022 [108]	To co-design a website to support diet and physical activity after stroke	Website prototype for eating well and moving more after stroke (i-Rebound Online)
Power, 2019 [109]	To develop an exoskeleton for lower limb assistance	Soft exoskeleton for lower limb assistance
Ramage, 2019 [110]	To use an integrated knowledge translation approach to co-design a physical activity intervention for people with stroke	Physical activity intervention for stroke survivors
Rosbergen, 2021 [111]	To use co-design to develop and test a telerehabilitation physical activity intervention for people with stroke	Telerehabilitation intervention to increase physical activity after stroke
Rose, 2017 [112]	To co-design an app that supports healthcare conversations	App to support hospital healthcare conversations
Ruddell, 2018 [113]	To evaluate the impact of a co-designed dietary intervention on an acute stroke unit	Multidisciplinary team rehabilitation service
Sadler, 2017 [114]	To describe the development and evaluation of a peer-support intervention to enhance resilience after stroke	Peer support intervention to promote resilience after stroke
Said, 2021 [115]	To use co-design to develop a shared decision-making tool to support telerehabilitation for people with moderate to severe disability after stroke	Shared decision-making tool to support the implementation of telerehabilitation for stroke survivors with moderate to severe disability.
Semprini, 2022 [116]	To identify the users’ needs and develop a control system for a lower limb stroke rehabilitation exoskeleton	Lower limb exoskeleton for stroke rehabilitation (TWIN exoskeleton; modifying it for people with stroke; initially designed for people with spinal cord injury)
Seregni, 2021 [56]	To describe the co-design process and evaluation of a virtual coaching system	Virtual coaching intervention for individuals with neurological conditions
Stewart, 2020 [117]	To describe the design of a behaviour change intervention that aims to enhance clinicians’ use of active practice strategies with patients in inpatient stroke rehabilitation	Behaviour change intervention to increase active practice during inpatient stroke rehabilitation
Teeling, 2019 [118]	To describe a co-designed process that will optimize patient access to mealtime assistance, decrease missed meal incidence, risk of malnutrition, reduce food waste and staff rework	Process for ensuring access to assistance at mealtimes
Termoz, 2022 [32]	To co-design and test a stroke transition program (protocol)	Stroke care transition intervention (Navistroke)
Thayabaranathan, 2022 [119]	To co-design and evaluate a yoga-based mindfulness intervention for people with stroke	Yoga-based mindfulness stroke intervention
Turton, 2017 [120]	To use co-design to inform the design of soft robotic trousers for improving mobility in older adults	Robotic trousers for improving mobility in older people, including people with stroke
VonKoch, 2022 [121]	To assess the feasibility of a care transition support co-designed with individuals with stroke, significant others, and healthcare professionals	Stroke care transition intervention
Wilkinson, 2011 [122]	To co-design self-management upper limb exercise rehabilitation technologies	Rehabilitation technologies to motivate patients to self-manage their upper limb exercise programme through the development of interactive computer-based systems
Wilson, 2015 [57]	To describe techniques for co-design with individuals with aphasia	Two tools: gesture therapy (GeST) and virtual speech practicing system (EVA Park)
Wu, 2019 [123]	To describe the design and development of a mobile health system for delivering secondary prevention of stroke	Mobile Health System for information on secondary prevention of stroke
Young, 2021 [124]	To conduct a patient public involvement activity to identify priority design features for power-assisted exercise equipment	Power-assisted exercise equipment for people with stroke
Young, 2021 [125]	To identify end-user perspectives (i.e., people with stroke and stroke professionals) on digital power-assisted exercise equipment and select priorities for prototype development	Power-assisted exercise equipment for people with stroke
Zacharia, 2021 [26]	To co-design a telehealth-based diet program for individuals with stroke	Mediterranean dietary telehealth intervention

### How co-design methods have been used to develop stroke interventions (Objective 2)

Based on our descriptive qualitative content analysis, we categorized the information in the articles according to the rationale for using co-design, types of co-designed stroke interventions, co-design participants, research methodologies/approaches to co-designing stroke interventions, methods of incorporating end-users in the research, co-design limitations, challenges, and potential strategies.

#### 1) Rationale for using co-design

Across the included data sources, researchers used co-design methods for several reasons and motivations, including to engage end-users in stroke intervention development and create changes in stroke services and within organizations.

Researchers used a co-design methodology to engage end-users in stroke intervention development. Co-design methodologies, such as experience-based co-design, offered researchers a structured but flexible and fluid approach to stroke intervention design [29, 49, 50]. Researchers indicated that the co-design products provided them with new ideas and concepts to understand how to manage and address complex conditions [104]. Researchers commonly used co-design to comprehensively understand end-user needs (e.g., individuals with stroke and stroke clinicians), prioritize solutions, and engage multiple end-users in developing stroke interventions [27, 43, 46, 48, 50, 51, 72, 98]. Moreover, researchers noted that by adopting a co-design methodology, interactions between multiple end-users were based on “partnership, equity and shared leadership” and could be used to produce creative, innovative and improve service design, management and evaluation [27, 43, 47–50].

The second commonly cited rationale by researchers for using co-design was that it could be an agent to create change. Researchers perceived co-design as a powerful agent for change in that it could help initiate change or priorities for change to improve stroke services [43, 50, 53, 124]. Co-designed interventions were believed to be more likely to be adopted because they integrated multiple end-users’ needs, abilities and challenges, thereby minimizing or even eliminating the misalignment between end-users and solutions designed and increasing the acceptability and feasibility of the co-designed intervention [26, 27, 35, 46, 48, 50, 51, 56, 65, 68, 85, 101, 107, 109, 125]. Through their involvement, end-users could identify the strengths and weaknesses of the stroke care system, paving the way for improvements to the quality of healthcare and end-users’ outcomes and satisfaction [35, 43, 46, 50, 56, 87]. In addition, co-design was seen as a vehicle for shifting the role of patients as receivers of health services to “experts by experience” [35, 50] and improving the content and delivery of the intervention [119].

At the organization level, researchers indicated that co-design could help them generate solutions grounded in the context, increasing organizational innovation capacity [32, 35]. Researchers also used co-design methods because these were successfully used to produce practical, accessible, and sustainable interventions and improve services in other healthcare settings (e.g., [49, 50, 62, 72]). Still, they were not widely used within specific stroke care settings, such as acute stroke services [49, 50, 62, 72]. Within one data source, researchers indicated that these methods could foster new and ongoing engagement and partnership processes between health organizations and end users [50].

#### 2) Types of co-designed stroke interventions

More than half (n = 58/89, 65%) of the co-designed interventions were technology-based, and most technology-based intervention studies (71%) were published between 2019–2023. Eight interventions were exclusively for people with aphasia (n = 8/88, 9%). The most common types of co-designed stroke interventions within the included data sources were: 1) physical rehabilitation or activity interventions (e.g., devices for upper limb rehabilitation; n = 42/89, 47%), 2) social/leisure interventions (e.g., peer support interventions; n = 10/89, 11%), 3) self-management interventions (e.g., goal setting resource; n = 11/89, 12%), 4) educational/informational interventions (e.g., discharge summary tool; n = 10/89, 11%), and 5) dietary interventions (e.g., meal time intervention; n = 6/89, 7%). Less common were interventions to support cognition (e.g., website for cognitive rehabilitation; n = 5/89, 6%), facilitate health communication (e.g., patient-provider pre-visit preparation tool; n = 4/88, 5%), fatigue management (n = 1/89, 1%), oral care management (n = 1/89, 1%), and visual therapy (n = 1/89, 1%).

Most co-designed stroke interventions were designed for use in community/home settings (n = 38/88, 43%), while fewer were to be used in inpatient settings (n = 14/89, 16%), during care transitions (n = 7/89, 8%) or across the care continuum (n = 8/89, 9%). However, 20 data sources (22%) did not clearly report a specific setting where the co-designed stroke intervention was intended.

#### 3) Types of co-design participants

Most data sources (n = 73/89, 82%) conducted co-design with multiple participant types (e.g., individuals with stroke, stroke family members/caregivers, stroke clinicians). Of the included data sources, nearly all (n = 87/89, 98%) involved people with stroke, and some specifically targeted individuals with aphasia (e.g., [43, 46, 57, 65, 67, 92, 94]), with a loss of visual field (e.g., [70]), current or former hospital inpatients (e.g., [32, 45, 49, 50, 66, 79, 86, 87]), with cognitive and/or physical impairments (e.g., [85]), with certain levels of physical or functional impairments (e.g., “severe upper-limb deficits” [91] and “mild to moderate level of disability” [55]), First Nations community members with stroke [64] and Aboriginal and/or Torres Strait Islander Peoples with stroke [43]. A few researchers specified that cognitive ability was determined through clinical judgement and/or based on whether an individual could provide informed consent and participate in the study activities [74, 104]. One study excluded individuals with “cognitive difficulties that made participation impossible, even in a small group discussion or together with next of kin” [51]. Studies assessed aphasia presence and/or severity through participant self-report, researcher observation [65, 73], a speech-language pathologist’s clinical judgement [46] and the Aphasia Severity Rating [46]. The Fugl-Meyer, Modified Ashworth Scale and Modified Rankin Scale were formal measures to assess participants’ upper-limb function [55, 91]. However, some studies had an eligibility requirement related to function, including the ability to ambulate short distances independently [74]. In contrast, another study included individuals with any type or level of physical impairment as it could help researchers determine which individuals could benefit from the intervention [77].

Co-design was also conducted with clinicians (n = 63/89, 71%) and family members/caregivers affected by stroke (n = 47/89, 53%). A quarter of included data sources (n = 22/89, 25%) conducted co-design with other types of end-users, such as managers, stroke foundations, government staff, stroke research experts, developers, designers, community members, academics, quality improvement experts, support staff, volunteers, engineers, technologists, and non-clinical staff (e.g., catering staff).

#### 4) Research methodologies/approaches to co-designing stroke interventions

The included data sources used a variety of research methodologies/approaches to co-design stroke interventions, with 21 data sources (24%) using multiple research methodologies/approaches. Commonly used research methodologies/approaches within the data sources included a ‘co-design process/approach’ or ‘co-design methodology’ (n = 21/88, 24%). A co-design approach included using focus groups to understand end-user needs and engage them in intervention development, while a co-design methodology was generally more structured (e.g., a four-phased methodology to explore, co-design, validate and develop). In addition to explicitly using co-design, data sources cited using other approaches, such as user (or human)-centred design (n = 14/89, 16%), experienced-based co-design (n = 13/89, 15%), participatory action research (n = 8/88, 9%), and participatory design/co-design (n = 9/89, 10%). Twelve (14%) data sources did not clearly indicate their research approach.

#### 5) Methods of incorporating end-users in the research

Workshops, interviews and/or focus groups were used as qualitative data collection methods in more than half of the included studies (n = 73/89, 82%) to gather data during the co-design stroke intervention phase. These methods helped researchers understand end-users’ needs and how they would engage with the solution, create an equal representation of end-user perspectives, and prioritize solutions. Focus groups were also conducted to create an environment of collaborative discussion between end-users and the research team, reflect on challenges, generate innovative ideas, and discuss potential solutions. Researchers also reported conducting separate focus groups with different end-users (e.g., clinicians and people with stroke), with some researchers bringing the end-users together at some point.

Prioritization was described as an essential part of co-design. Various approaches were used across studies to reach a consensus on priorities for the co-designed stroke intervention, such as focus group discussions and end-users ranking or voting using surveys or other engagement tools (e.g., [29, 35, 43, 45, 49, 65, 79, 94, 109]). Within one study, people with stroke had the final say in resource design if the prioritization results differed between the perspectives of a clinician and a person with stroke [65].

#### 6) Co-design limitations, challenges and potential strategies

The limitations and challenges reported within the included data sources and potential strategies that may help overcome the limitations and challenges were organized into five subcategories: 1) recruitment-related, 2) participant-engagement, 3) contextual and logistical, and 4) ethical (see Table 2). While some of the identified limitations, challenges and strategies relate to co-design methods broadly, below, we have focused on the information that our research team determined was most relevant to our co-designing stroke interventions to align with the focus of this review.

**Table 2:** Summary of co-design limitations and challenges and potential strategies to overcome these limitations and challenges.

Limitations and challenges reported within included studies	Potential strategies to overcome identified limitations and challenges generated from the methods used within included studies
**Recruitment-related** • Difficulties recruiting participants resulting in small sample sizes • Low diversity within recruited participants (e.g., age, cognition, function, language fluency, etc.) • Difficulties assessing cognitive or functional status **Participant-engagement** • Success of prior co-design stage/phase can be dependent on the previous stage/phase • Preconceptions and differing levels of topic knowledge or interest • Burden on participants • Different levels of ability required to participate in activities and provide feedback • Different participant priorities and levels of enthusiasm for the project • Ambiguous roles of participants • Power dynamics among participants as well as between participants and researchers • Presence of the researcher may have limited participants’ participation • Unclear/poorly defined assignments and methods can negatively affect equal and meaningful participation **Contextual and logistical** • Not all participants had access to technology for remote data collection during COVID-19 pandemic • Time and resource constraints can limit the iterative process • Considerations for implementation include costs, roles, and feasibility • Location and time of data collection • Differing organizational priorities and levels of support **Ethical considerations** • Participant burden • Obtaining informed consent from people with different abilities (e.g., those with cognitive impairments)	**Recruitment-related** • Advisory groups or clinical champions may support recruitment and optimize the acceptability and relevance of interventions • Purposeful recruitment to ensure diverse participant perspectives are represented (e.g., equal numbers of clinicians and patients) **Participant-engagement** • Structured methodology with deliverables and key observations to ensure design/redesign meaningfully captures participant perspectives • Acknowledgment that co-designed products can result in improved quality of stroke care • Iterative co-design to include multiple perspectives • Explore the utility of structured but accelerated co-design methods if time and resource constraints are a concern • Multidisciplinary researchers should articulate expectations and assumptions early in the project and collaboratively build data collection tools and conduct analysis • Use of personas or characters to create empathy • Multiple small groups, grouped by participant type • Managing participant expectations • Evidenced-base communication strategies • Speech-language pathologist or trained facilitator • Facilitation by people with stroke and without the researcher • Facilitators should be skilled in handling different perspectives, and the research team should determine how to select, prioritize and balance differing (including conflicting) participant perspectives in the design • Pilot test methods and conduct facilitator training based on pilot test • Ensure accessible meeting rooms and activities • Use of different tools to engage people with stroke who can have different levels of communication skills in co-design **Contextual and logistical** • Steering committee to monitor timely project completion • Build in time for the research team conduct pre-work (e.g., literature reviews) prior to data collection to maximize efficiency/outputs during data collection with participants • Gain leadership support for project/project activities e.g., include clinical leads on research team members to build project support, provide regular project updates, highlight tangible benefits for an organization • Create a space where participants can feel like equal co-design group members (e.g., participants bring to physical space their familiar home items, creating a relaxed, easy and comfortable atmosphere) • Data collection with clinicians should not conflict/interfere with clinical care duties • Evaluate the impact of change from co-designed intervention to demonstrate its benefits • Highlight tangible benefits to the organization or community **Ethical Considerations** • Ensure adequate participant reimbursement to reduce participant burden (e.g., reimburse transportation costs, accommodations, meals) • Allow participants to review material that uses their image/voice (e.g., film narratives) • Ethical checks and processes integrated into the project to ensure participants are fully informed of co-design activities

#### Subcategory 1) Recruitment-related limitations, challenges and strategies

In terms of recruitment-related limitations and challenges, many researchers reported difficulty recruiting participants with stroke and clinicians (e.g., [26, 45, 48, 51, 55, 60, 65, 104, 116, 126]). Reasons for recruitment challenges included participant burden associated with high time commitments for activities [65], limited technological access for participation during the COVID-19 pandemic [51], and time and resource constraints for conducting the co-design study and accommodating end-users’ differing needs (e.g., [26, 53, 55, 56, 107, 116]). Individuals with stroke declined to participate in studies due to poor health and lack of perceived benefit [114] or were ineligible to participate in some studies due to certain cognitive, communication or functional impairments (e.g., [51, 74, 78, 104]). One of the limitations of a small sample size was the reduced diversity of participant perspectives within they study (e.g., a lack of representation of participants from underrepresented groups, including low socioeconomic backgrounds, lower levels of completed education, and cultural minority groups [65]). Researchers also noted that the transferability of their results could have been impacted by the lack of perspectives from multiple groups of end-users, such as patients [60, 97], family members [97], certain types of health professionals, including occupational and speech therapists [26, 97], individuals with different cognitive and functional abilities [85, 104], and people at different stages in their stroke journey [27, 46, 60].

In terms of recruitment strategies, some researchers used targeted recruitment strategies to recruit a diverse sample of participants. For instance, to recruit individuals from First Nations communities, researchers co-developed a recruitment video with the Indigenous community [43] and sought additional ethics approval and appointments: “Aboriginal research engagement and ethics approvals; appointment of an Aboriginal Project Manager and two trained Aboriginal group facilitators” [61].

#### Subcategory 2) Participant-engagement limitations, challenges and strategies

In terms of participant-engagement limitations and challenges, some participants had different levels of knowledge about the study topic or cognitive/communication challenges that required additional time and resources from the researchers to provide education, confirm that participants understood the activities [43, 98, 125], and verify that researchers accurately understood the information shared by participants [54]. For instance, researchers made time to verbally discuss the research process before data collection sessions with participants [43], and many researchers created materials in accessible formats and distributed these to participants before synchronous data collection to support meaningful participant engagement in co-design [26, 43, 65].

Some individuals with stroke struggled to attend or meaningfully engage in each co-design activity due to fatigue, medical appointments, poor health, and lack of public transportation to accommodate stroke-related disabilities [114]. Also, inaccessibility to technology for remote data collection [27, 51] and varying levels of physical and cognitive ability amongst individuals to partake in activities and provide feedback (e.g., [48, 57, 65, 89]) prevented participants from completing the co-design activities. For example, individuals with aphasia after a stroke or stroke-related physical disabilities reported difficulties with completing written and lengthy surveys [65, 114]. In addition, individuals with physical impairments (e.g., manual dexterity challenges) could not participate in a planned design activity [57]. Post-stroke memory issues also may have affected follow-up and led to missing data [114]. Moreover, due to the health of some individuals with stroke, researchers who facilitated the co-design activities had to consider the well-being of these participants and the possibility of tending to unexpected situations, such as feeling faint during games or physical activities [54].

Challenges with group dynamics were commonly reported and hindered meaningful participation in activities [52, 95, 96, 123, 125]. Power imbalances among participants were identified between stroke clinicians and patients and their caregivers, with risks that patients and caregivers may not perceive themselves as experts compared to clinicians [52]. Hierarchies were also present in acute stroke and rehabilitation units amongst multidisciplinary team members, and limited participation in focus groups was reported due to fear of speaking out about issues on the unit [117]. Moreover, participants and researchers sometimes had diverging priorities, which could result in disagreement and negatively affect collaboration [123, 125]. The reduced level of participant engagement was also influenced by the presence of the researcher [50, 51, 117] and the ambiguous roles of participants in the research process [96].

In terms of participant-engagement strategies, researchers used various methods and tools to engage participants in co-design during the interviews and focus groups, including film narratives, personas, maps (e.g., patient journey, emotional or behaviour maps) and other strategies (see Table 3). These engagement tools/strategies were used by researchers to create a space for participants that allowed participants to share their perspectives during the co-design, as researchers indicated that the goals of co-design were to create “mutual trust, and a sense of communal action to change” [29] and “co-learning and capacity building” [63]. Prior to data collection, many researchers developed recruitment and data collection activities, questions and prompts with individuals with stroke (e.g., an advisory committee) or pilot tested these with individuals who had a stroke to ensure these were appropriate (e.g., could enable discussion and agreement and allow individuals with stroke decision-making power [65]) and accessible (e.g., delivery format to accommodate differing communication needs (e.g. [43, 54, 65, 73]) for people with stroke (e.g., an advisory board and/or each participant), as well as feasible (e.g., time to completion fell within the allotted time) (e.g., [32, 116]). In addition, some researchers hired experts, including an animator, speech-language pathologist, graphic designer, and professional voice recording artist, to create generative materials to develop data collection tools to help meaningfully involve participants and elicit rich dialogue (e.g., [43, 46, 65]). Researchers used multimodal communication to collect data from participants with different cognitive, functional and communication abilities, including writing out keywords and using fixed choice questions or open-ended questions with prompts, and visual approaches to capture participant thoughts (e.g., gestures, cards depicting emotions to convey perspectives, story grids, photo diaries, prototypes, commentary charts or participants could select “coloured cards depicting happy, neutral, and sad faces to represent their opinion” [65]) [43, 54, 57, 65, 73]. In addition, researchers noted that it was necessary to provide sufficient processing time for participants to comprehend and generate a response [43], be aware or refrain from assigning physical tasks (e.g., making an artifact) [54], be mindful of post-stroke fatigue [45], and verify content with participants to ensure researchers accurately interpreted the data [43]. Some studies used trained facilitators, such as speech-language pathologists, psychologists, nurses or others trained in supportive communication strategies to facilitate or co-facilitate data collection to support communication with participants who had aphasia [43, 54, 65, 73] or included caregivers or significant others to support communication [73]. In one study, researchers used a structured focus group technique called the nominal group technique to support participation in group discussions by all members and provide supportive communication strategies for people with communication impairments [43]. The researchers indicated that this technique had been used in prior studies with people with aphasia [43]. In addition, researchers consulted prior literature to determine the optimal length and group size for data collection (e.g., 90-minute co-design workshops with 4–5 participants [43]). Finally, some researchers included a validation phase to confirm their understanding and interpretations of participant comments (e.g., inviting feedback on researcher notes, ideas, and potential solutions) [27, 65].

**Table 3:** Examples of methods or tools used during co-design.

Examples of methods or tools used during co-design	Examples of how methods or tools were used within included data sources
Someone who isn’t me [57]	Participants were asked to share what they thought the perspectives/thoughts of a friend with aphasia would be or if they had severe aphasia
Ice breaker games [57]	Conducted at the start of the group session to break the ice
Observations and field notes [29, 43, 45, 47, 49, 50, 52, 60, 66, 85–89, 94, 96, 116–118, 119, 127, 128]	Various observations of participants to gain contextual insights and identify key considerations
Story Grids technique (adaptation of talking Mats^TM^) [57]	Participants could express their opinions using symbols or photos rather than verbally
Photo diary [57]	Participants captured situations in their everyday lives where they encountered communication challenges
Film narratives or trigger films (created using participant experiences and voices) [29, 35, 43, 45, 49, 50, 95]	Films displayed patient experiences, demonstrated commonalities and differences among experiences, created shared understanding and sense of cohesiveness/collective action, validated key issues, and identified priorities for change
Personas: “represents a member of a future user group, or a set of prototypes that offer alternative solutions for a digital tool” [26, 35, 43, 51, 63, 85]	Created to “represent users of different sexes, ages, personalities, life situations, values, and interests” [51] and “different genders, employment status, support structures and stroke outcomes” [26] Created to represent common stroke-related impairments Used to facilitate discussion, problem-solving and idea generation
Patient journey/journey mapping [43, 96, 118]	Map patient experiences and interactions within health service to understand processes and identify needs
Lego® Serious Play® method [27]	Participants create a Lego® model to answer the question. This method was intended to promote in-depth group discussions, understand participant experiences, needs and potential solutions
Emotional mapping [29, 50] or visual analog mood scales [94]	Mapping the emotional journey of a patient during a period to understand the problem and inform improvement priorities
Behavioural mapping [29, 47, 49, 50, 87]	Used to understand problems (e.g., identify patients’ social, cognitive or physical activity)
Participant-reported outcomes (e.g., questionnaires/surveys) [47, 49, 51, 52, 56, 65, 66, 69, 72, 76, 82, 87, 89, 91, 93, 96, 97, 116]	Identify participant needs, priorities and evaluate solutions
Nominal group technique [43]	Used to gather, understand, and prioritize data and enable supportive communication techniques/strategies for people with communication impairments
Design-by-playing [54]	Used to encourage participants’ expression of ideas in different ways

According to researchers, group-based co-design sessions/activities were intended to be conducted with participants who had “equal cognitive and physical abilities [to] participate in the development process” [55, 98]. Thus, researchers separated data collection groups by end-users (e.g., clinicians and people with stroke) [26, 27, 45], including separate aphasia-friendly workshops for people with stroke [108]. Data collection activities for clinicians included surveys and questionnaires to minimize time commitments [29].

#### Subcategory 3) Contextual and logistical considerations

Time and funding constraints limited the iterative co-design process, as additional workshops, meetings, prototypes, or evaluations for further data collection could not occur without additional resources and time [26, 95, 109], which prevented optimal stroke intervention from being created to meet the needs and priorities of participants [109]. It was resource-intensive for researchers to design engaging co-design activities, consider the various needs of participants to improve accessibility and participation, and coordinate meetings and workshops during the data collection [50]. Another challenge reported was that in co-design studies that utilize multiple phases, the failure of one phase could influence the outcomes of the next phase and limit results [72].

In addition, researchers noted that factors that reduced engagement in co-design activities, such as clinicians faced scheduling conflicts [72] and organizational constraints, such as short staffing and high workloads [45, 66], which limited the amount of data gathered, and the interpretations made [27]. Another logistical challenge was the differing priorities between the organization and the researchers, where the organization wanted to focus more on service improvement rather than developing a new program [66] and when suggested changes were not possible (e.g., altering essential components of an intervention) [65]. Finally, some researchers raised the need to evaluate the future sustainability of the co-designed intervention (e.g., [50, 63]), which may be impacted by contextual factors such as how teams work.

In terms of contextual and logistical strategies, as co-design was an added workload to staff clinical duties, managerial encouragement and support were essential for clinicians to participate [50]. To minimize participant burden, researchers scheduled data collection at times and places most convenient to participants [29, 65]. For instance, researchers conducted focus groups during program sessions that were part of participants’ everyday routines to reduce time and travel burdens for participants and caregivers [65]. The most convenient place for clinicians may be in the hospital or other clinical settings, but it should not interfere with their clinical duties [29]. More than half of the included data sources (n = 52, 59%) reported an evaluation of the interventions (e.g., sustainability, resources, and feasibility of implementation). Finally, due to the COVID-19 pandemic, some researchers completed online co-design sessions (e.g., [46, 94]).

#### Subcategory 4) Ethical challenges and strategies

Ethical challenges included obtaining consent from individuals with communicative or cognitive impairments due to stroke-related impairments and excessive participant burdens [45, 49, 50].

In terms of ethical strategies, some researchers included individuals with cognitive or individuals with different communicative, physical or cognitive abilities by providing accommodations to attain informed consent [46]. For example, a psychologist trained in supportive communication strategies for people with aphasia was involved in the informed consent for participants with communication challenges (e.g., {Anemaat, 2021 #41}). Researchers tailored information to enhance communicative accessibility for individuals with aphasia following aphasia-friendly principles (e.g., large font, pictures, verbal reading forms, yes/no verification techniques, and simple sentences) [26, 43, 72]. Researchers accepted a witnessed mark or line for written informed consent in the place of a signature from those with post-stroke physical challenges using their dominant arm [72]. However, some studies excluded individuals with cognitive or communication challenges due to ethical concerns (e.g., [51, 74, 78, 104]).

## Discussion

The findings of this review expand prior knowledge regarding co-designed stroke interventions through a comprehensive synthesis of the diverse co-design methodologies employed by researchers. This synthesis of diverse publication types also provides insights into the challenges and limitations of co-design methodologies and potential strategies to overcome these challenges in using co-design.

Through this scoping review, we aimed to map the extent of existing research that has used co-design for stroke intervention development and describe how it was used. We identified 219 data sources with methods aligned with the broad definition of co-design adopted in this review. However, variable terminology was used to describe the co-design methodologies. Also, significant variations in end-user engagement in the design process were noted throughout the studies. The varying levels of engagement posed differing limitations and challenges encountered, creating a need for a future guide to identify the appropriate level of engagement. From a methods perspective, future co-design studies should clearly indicate their research approach, details of the co-design activities, and how different perspectives and solutions were prioritized.

In line with prior literature [8, 10], there are challenges associated with the use of codesign approaches, some of which are specific to codesign and others more general to research focused on stroke. Engaging in co-design research requires adequate resources, particularly time and budget, to engage people with stroke in effectively and meaningfully co-designing stroke interventions. The iterative nature of co-design work can make it challenging to plan methods in advance, as the success of one stage can depend on the previous stage [129]. When considering issues related to research with a stroke population, it is important to note that a substantial portion of individuals with stroke live with lasting post-stroke impairments, spanning motor, cognitive, sensory and communicative domains [130, 131]. Stroke research studies, however, [51, 56, 74, 78, 104]) have tended to exclude individuals with physical, communicative or cognitive impairments, limiting the generalizability and breadth of knowledge acquired due to the limited representativeness of the typical stroke population [131–134]. Common reasons for excluding individuals with physical, communicative, and cognitive impairments include limitations in the accessibility of planned data collection activities and troubles obtaining informed consent [50, 77, 135]. Since a significant number of individuals with stroke who would benefit from these interventions would not meet certain eligibility criteria, researchers should consider strategies to enhance equitable access to research opportunities [136]. One approach to accomplish increased accessibility could include researchers identifying the needs of the participants before data collection and tailoring the activities accordingly, rather than selecting participants suitable in the level of function or skills needed to engage in the pre-planned activity. The use of flexible methods, such as tailoring data collection to align with participants’ needs, allows researchers to successfully include people with stroke who had post-stroke challenges by investing the time and resources required to engage with them meaningfully for data-collection [43, 46, 57, 65, 67, 70, 92, 94].

Co-design methodologies commonly use workshops, interviews, and focus groups for data acquisition methods, with end-users taking on different roles. However, meaningfully engaging end-users in co-design can be challenging [8, 12]. We synthesized strategies that may help reduce or mitigate the challenges and limitations identified by the included studies (Table 2). Adopting these strategies may improve the success of co-design for stroke intervention development in future studies. For example, tailoring data collection to meet the needs of individuals with stroke is necessary because individuals with stroke have different needs (e.g., being mindful of participants’ function when assigning physical tasks) [54, 129, 130]. Also, if individualized and tailored approaches to stroke care are common in clinical practice (e.g., [137–140]), these approaches should be adopted for research and data collection [141]. Researchers will need to allocate sufficient time (e.g., meeting with individuals before data collection to understand how to engage them in data collection meaningfully) and resources (e.g., accessible written communication) to tailor data collection activities to meet the needs of individuals with stroke [26, 43, 65]. When designing co-design activities, researchers should consider how to engage individuals with stroke who have aphasia, challenges reading due to literacy, and visual and perceptual impairments in co-design [142]. Preparation, through consultation with the end-users, is needed to maximize the outcomes of co-design activities [143]. In addition, generative tools, such as Lego™, videos, and emotional mapping, can help engage individuals with communication difficulties to express themselves visually and verbally to describe potential solutions [144].

Up to 38% of people with stroke have aphasia [145]. Consequently, exploring personalized techniques to enhance engagement among individuals with post-stroke aphasia is highly significant for co-design success. Researchers may need to identify participants’ communication profiles to conduct co-design sessions involving individuals with post-stroke aphasia effectively. While previous studies have highlighted the need for facilitators skilled in engagement, communication, and facilitation [12], we highlight the additional need for facilitators to be skilled in communication with people who have had a stroke, including those with cognitive or communication impairments. If a research team lacks these skills, resources may be required to hire a skilled facilitator, including speech-language pathologists or other qualified personnel [43, 54, 65, 73]. While some researchers may rely on caregivers/family members for communication support [73], due to the burden on caregivers and potential differences in the participant and caregiver/family members’ responses, it is recommended that data collection methods be modified to be more accessible [146]. Another consideration for co-design with individuals with stroke is the participants’ informational and literacy needs; this may require a need to account for additional time in developing written materials to ensure the materials are at appropriate reading levels (e.g., [147–149]). These strategies may apply to other groups with communication challenges.

As noted in this review, co-design can be burdensome for the end-users due to its iterative nature and participant involvement over variation in time (i.e., it is not usually a one-time data collection process) [43]. Researchers need to integrate strategies to reduce the burden on participants for enhanced participation. For example, consistent with prior literature [12, 150], studies in this review noted many challenges involving clinicians in co-design, including a lack of time and a busy workload [151]. Strategies used by researchers in our review included adapted or accelerated approaches to co-design [45, 69] and the use of asynchronous data collection [29] or providing travel reimbursements [43]. In addition, some researchers conducted virtual co-design due to the COVID-19 pandemic [46, 51, 94]. While virtual co-design has benefits, such as reducing the impact of location and functional and cognitive disability on participant engagement in co-design and may require fewer resources [152, 153], researchers must consider the unique challenges of conducting co-design virtually, particularly if participants have low digital skills or have cognitive, communication and/or visual challenges [149, 153, 154]. Additionally, some participants may not have the same access to reliable internet and adequate technology [155], leading to inequitable opportunities to participate. Also, ethical issues, such as informed consent capacity and data validity, can be magnified during virtual data collection [149]. Virtual co-design may reduce participant burden (e.g., travel) [152, 153]; however, there are few models for people with stroke, particularly people with aphasia. In addition, researchers noted inclusion limitations with virtual-only data collection, such that people who do not have access to technology, are not comfortable with it, or are unable to use the virtual platforms would be excluded [153, 156]. Models of hybrid co-design may be explored in future research [157], allowing in-person and online co-design sessions for end-users to choose which format would be best for them. A hybrid co-design model may reduce the resources required for co-design (e.g., participant time/travel costs) and allow for greater accessibility, but this has not been evaluated yet. Future research endeavours may need to compare the effectiveness and utility of various co-design approaches, including in-person, online and hybrid co-design methods. While some studies in our review indicated they conducted integrated methods to prioritize differing participant needs/suggestions, few details were reported on how they prioritized solutions and managed conflicts during co-design sessions. Effective prioritization requires consideration of the project goal and what is possible within the constraints of the project. For instance, one study indicated that people with stroke had the final say in resource design if the prioritization results differed between the clinician and a person with stroke [65]; however, this may result in the loss of a critical implementation perspective of clinicians. Another study discussed various approaches to prioritization [158]. They first explore quantitative group rankings as a common method; however, reaching consensus through ranking will often lead to less individuality and subtlety of the topic. Other approaches discussed include facilitated discussions and including the end-users as contributors to the analysis stage of the process [159, 160]. Their study utilized a combination of quantitative ranking with a subsequent facilitated discussion.

Finally, few studies evaluated their co-design methodology (e.g., impact on co-design participants, health programs, and organization) [161]. It may be valuable for researchers to continuously assess whether their co-design processes are working well and whether adjustments may be needed. For instance, Wilson and colleagues integrated participant feedback to refine and pivot their co-design activities to ones that would work better for their specific group [57]. This ongoing evaluation involved researchers reflecting on their methods and refining them to successful participant engagement [57], which may be particularly helpful when co-designing with complex populations. Evaluating the impact of methodology effectiveness may also help researchers decide if the co-design methodology employed and invested in is worthwhile for their specific project.

### Strengths and limitations

Several strategies were implemented to reduce the risk of missing relevant studies, including developing a comprehensive peer-reviewed search strategy in consultation with an experienced librarian, pilot testing our inclusion criteria before initiating title and abstract screening, having two research team members independently screen each data source to ensure a strong understanding of the inclusion criteria, and reviewing the reference list search of 10 articles to identify relevant studies that may have been missed. Moreover, since researchers may classify their approach with methodologies associated with co-design (e.g., co-produce, co-creation) we used a broad definition of co-design to capture these studies. However, it was difficult to ascertain the level of overlap with co-design [25]. For this reason, we limited our in-depth analysis to studies that explicitly used the term co-design. Per our protocol, a consultation activity will be conducted and published elsewhere to identify further considerations for co-designing interventions with people with a stroke that these findings have not captured.

Despite these efforts, we may not have identified all data sources meeting inclusion criteria due to search limitations, which allow only keyword searching in a title and abstract and human errors during screening (e.g., excluding studies that were unclear if they used co-design). As authors may use interchangeable terms related to co-design, with the underlying principles overlapping significantly, the current review is not a comprehensive synthesis of all participatory methods being used by stroke researchers within intervention development. Future reviews should synthesize literature on all participatory methods to identify the similarities and differences between various methods and/or if further challenges and strategies can be identified from the literature. Second, since grey literature was captured within our database search, we did not conduct an additional search for grey literature. Third, given the growing use of codesign, a future review with an updated search may be necessary since our database search conducted on December 20, 2022 may become outdated due to increasing interest in co-design methodologies. Finally, we did not search the PeDro database since it does not have comprehensive search capabilities.

## Conclusions

This scoping review has identified a broad range of literature using co-design and associated methodologies to develop stroke interventions. This review can inform future studies seeking to co-design a stroke intervention by providing a background on how co-design methodology has been used and can be tailored to include individuals with stroke with different communication, functional and cognitive abilities.

## Supporting information

S1 Appendix Database search strategies. (DOCX)

S1 Table List of all data sources that used co-design for stroke intervention development, including data sources that aligned with co-design or associated methodology with and without explicitly using the term co-design. (DOCX)

S1 File Preferred Reporting Items for Systematic reviews and Meta-Analyses extension for Scoping Reviews (PRISMA-ScR) checklist. (PDF)
