# Supplementary material for: Co-design for stroke intervention development: Results of a scoping review
Source: PLoS One. 2024 Feb 14;19(2):e0297162. doi: 10.1371/journal.pone.0297162 (PMC10866508; doi:10.1371/journal.pone.0297162)
Supplement: S1 Appendix — (DOCX) [file pone.0297162.s001.docx]

**Supporting information 1**

Database Search Strategies Designed By: Julia Martyniuk

Peer Reviewer: Ana Patricia Ayala

Other notes of search strategy construction:

The search was also limited to exclude animal only studies, and included only English language articles.

Searches were run on December 20, 2022. The strategies below correspond with the original search run on December 20, 2022.

Database(s): **Ovid MEDLINE: Epub Ahead of Print, In-Process & Other Non-Indexed Citations, Ovid MEDLINE® Daily and Ovid MEDLINE®**1946-Present
Search Strategy:

| **#** | **Searches** | **Results** |
| --- | --- | --- |
| 1 | "intracranial embolism and thrombosis"/ or intracranial embolism/ or exp intracranial thrombosis/ or exp intracranial hemorrhages/ or stroke/ or brain infarction/ or brain stem infarctions/ or lateral medullary syndrome/ or cerebral infarction/ or cadasil/ or dementia, multi-infarct/ or infarction, anterior cerebral artery/ or infarction, middle cerebral artery/ or infarction, posterior cerebral artery/ or hemorrhagic stroke/ or ischemic stroke/ or embolic stroke/ or thrombotic stroke/ or stroke, lacunar/ | 248018 |
| 2 | cerebrovascular disorders/ or hypoxia-ischemia, brain/ | 54493 |
| 3 | Stroke Rehabilitation/ | 16835 |
| 4 | (stroke* or poststroke* or transient ischemic attack*).tw,kf. | 309648 |
| 5 | ((cerebrovasc* or cerebr* vasc*) adj3 (accident* or disorder* or disease* or event* or incident* or infarct*)).tw,kf. | 49844 |
| 6 | ((cerebral or brain* or intracerebral or subarachnoid or "sub arachnoid" or intracranial or "intra cranial" or subdural or "sub dural" or cerebell* or vertebrobasil* or hemispher* or infratentorial or supratentorial or "middle cerebr*" or mca* or "anterior circulation") adj3 (infarct* or haemorrhag* or hemorrhag* or ischemia* or ischaemia* or bleed* or thrombos* or thrombus* or embolism* or embolus or embolitic or occlus* or hypoxi*)).tw,kf. | 164042 |
| 7 | 1 or 2 or 3 or 4 or 5 or 6 | 531480 |
| 8 | Community-Based Participatory Research/ | 5471 |
| 9 | community participation/ or patient participation/ | 46960 |
| 10 | Patient-Centered Care/ | 22512 |
| 11 | (codesign* or co-design*).tw,kf. | 2934 |
| 12 | (design* adj1 (particip* or experience based or person* based)).tw,kf. | 6076 |
| 13 | (action adj1 research).tw,kf. | 5561 |
| 14 | ((Patient* or user* or consumer* or stakeholder* or public* or client* or clinician* or provider* or caregiver* or communit* or inpatient* or outpatient* or people* or person* or individual*) adj1 (centered or centred or centric or participa* or engag* or empower* or involv* or voice or view or perspective or experience* or advoca* or behav* or evaluat* or communicat* or collaborat* or opinion* or input* or consult* or partner* or develop* or design* or dialog*)).tw,kf. | 512862 |
| 15 | (coproduc* or co-produc* or cocreat* or co-creat* or cocare or co-care or cocaring or co-caring or cocared or co-cared or cocommiss* or co-commiss* or Co-decide or co-decision* or co-decided or Codeci* or Co-deliver* or Codeliver* or co-evaluat* or Coevaluat* or Co-implement* or Coimplement* or Co-construct* or Coconstruct* or Co-innovat* or Coinnovat* or Co-learn* or Colearn*).tw,kf. | 14926 |
| 16 | 8 or 9 or 10 or 11 or 12 or 13 or 14 or 15 | 580662 |
| 17 | 7 and 16 | 16293 |
| 18 | Animals/ not Humans/ | 5041579 |
| 19 | 17 not 18 | 16228 |
| 20 | limit 19 to english language | 15331 |

Database(s): **Embase Classic+Embase**1947 to 2022 December 19
Search Strategy:

| **#** | **Searches** | **Results** |
| --- | --- | --- |
| 1 | brain embolism/ | 8830 |
| 2 | occlusive cerebrovascular disease/ or middle cerebral artery occlusion/ | 34607 |
| 3 | cerebrovascular accident/ or brainstem stroke/ or cardioembolic stroke/ or exp experimental stroke/ or exp ischemic stroke/ or lacunar stroke/ | 289513 |
| 4 | Wallenberg syndrome/ | 1114 |
| 5 | brain infarction/ or exp anterior circulation infarction/ or brain infarction size/ or brain stem infarction/ or cadasil/ or lacunar infarction/ or migrainous infarction/ or multiinfarct dementia/ or posterior circulation infarction/ | 85956 |
| 6 | cerebral artery disease/ | 5962 |
| 7 | brain hemorrhage/ or brain ventricle hemorrhage/ or cerebellum hemorrhage/ or massive intracerebral hemorrhage/ or exp subarachnoid hemorrhage/ | 175555 |
| 8 | hypoxic ischemic encephalopathy/ | 10141 |
| 9 | stroke rehabilitation/ | 6406 |
| 10 | (stroke* or poststroke* or transient ischemic attack*).tw,kf. | 499920 |
| 11 | ((cerebrovasc* or cerebr* vasc*) adj3 (accident* or disorder* or disease* or event* or incident* or infarct*)).tw,kf. | 78868 |
| 12 | ((cerebral or brain* or intracerebral or subarachnoid or "sub arachnoid" or intracranial or "intra cranial" or subdural or "sub dural" or cerebell* or vertebrobasil* or hemispher* or infratentorial or supratentorial or "middle cerebr*" or mca* or "anterior circulation") adj3 (infarct* or haemorrhag* or hemorrhag* or ischemia* or ischaemia* or bleed* or thrombos* or thrombus* or embolism* or embolus or embolitic or occlus* or hypoxi*)).tw,kf. | 243781 |
| 13 | 1 or 2 or 3 or 4 or 5 or 6 or 7 or 8 or 9 or 10 or 11 or 12 | 855879 |
| 14 | participatory research/ | 6791 |
| 15 | community participation/ | 3872 |
| 16 | patient participation/ | 33780 |
| 17 | (codesign* or co-design*).tw,kf. | 3549 |
| 18 | (design* adj1 (particip* or experience based or person* based)).tw,kf. | 7003 |
| 19 | (action adj1 research).tw,kf. | 6664 |
| 20 | ((Patient* or user* or consumer* or stakeholder* or public* or client* or clinician* or provider* or caregiver* or communit* or inpatient* or outpatient* or people* or person* or individual*) adj1 (centered or centred or centric or participa* or engag* or empower* or involv* or voice or view or perspective or experience* or advoca* or behav* or evaluat* or communicat* or collaborat* or opinion* or input* or consult* or partner* or develop* or design* or dialog*)).tw,kf. | 783053 |
| 21 | (coproduc* or co-produc* or cocreat* or co-creat* or cocare or co-care or cocaring or co-caring or cocared or co-cared or cocommiss* or co-commiss* or Co-decide or co-decision* or co-decided or Codeci* or Co-deliver* or Codeliver* or co-evaluat* or Coevaluat* or Co-implement* or Coimplement* or Co-construct* or Coconstruct* or Co-innovat* or Coinnovat* or Co-learn* or Colearn*).tw,kf. | 17168 |
| 22 | 14 or 15 or 16 or 17 or 18 or 19 or 20 or 21 | 838034 |
| 23 | 13 and 22 | 32379 |
| 24 | Animals/ not Humans/ | 1435341 |
| 25 | 23 not 24 | 32366 |
| 26 | limit 25 to english language | 31080 |

Database(s): **APA PsycInfo**1806 to December Week 2 2022
Search Strategy:

| **#** | **Searches** | **Results** |
| --- | --- | --- |
| 1 | cerebrovascular accidents/ or cerebrovascular disorders/ or cerebral hemorrhage/ or exp cerebral ischemia/ or subarachnoid hemorrhage/ | 31523 |
| 2 | (stroke* or poststroke* or transient ischemic attack*).tw. | 40072 |
| 3 | ((cerebrovasc* or cerebr* vasc*) adj3 (accident* or disorder* or disease* or event* or incident* or infarct*)).tw. | 5502 |
| 4 | ((cerebral or brain* or intracerebral or subarachnoid or "sub arachnoid" or intracranial or "intra cranial" or subdural or "sub dural" or cerebell* or vertebrobasil* or hemispher* or infratentorial or supratentorial or "middle cerebr*" or mca* or "anterior circulation") adj3 (infarct* or haemorrhag* or hemorrhag* or ischemia* or ischaemia* or bleed* or thrombos* or thrombus* or embolism* or embolus or embolitic or occlus* or hypoxi*)).tw. | 13594 |
| 5 | 1 or 2 or 3 or 4 | 53251 |
| 6 | community involvement/ | 6202 |
| 7 | client participation/ | 2961 |
| 8 | patient centered care/ | 526 |
| 9 | (codesign* or co-design*).tw. | 977 |
| 10 | (design* adj1 (particip* or experience based or person* based)).tw. | 4796 |
| 11 | (action adj1 research).tw. | 10077 |
| 12 | ((Patient* or user* or consumer* or stakeholder* or public* or client* or clinician* or provider* or caregiver* or communit* or inpatient* or outpatient* or people* or person* or individual*) adj1 (centered or centred or centric or participa* or engag* or empower* or involv* or voice or view or perspective or experience* or advoca* or behav* or evaluat* or communicat* or collaborat* or opinion* or input* or consult* or partner* or develop* or design* or dialog*)).tw. | 230519 |
| 13 | (coproduc* or co-produc* or cocreat* or co-creat* or cocare or co-care or cocaring or co-caring or cocared or co-cared or cocommiss* or co-commiss* or Co-decide or co-decision* or co-decided or Codeci* or Co-deliver* or Codeliver* or co-evaluat* or Coevaluat* or Co-implement* or Coimplement* or Co-construct* or Coconstruct* or Co-innovat* or Coinnovat* or Co-learn* or Colearn*).tw. | 9221 |
| 14 | 6 or 7 or 8 or 9 or 10 or 11 or 12 or 13 | 255698 |
| 15 | 5 and 14 | 2487 |
| 16 | Animals/ not Humans/ | 7411 |
| 17 | 15 not 16 | 2487 |
| 18 | limit 17 to english language | 2360 |

EBSCO CINAHL

| **#** | **Query** | **Limiters/Expanders** | **Last Run Via** | **Results** |
| --- | --- | --- | --- | --- |
| S20 | S17 NOT S18 | Narrow by Language: - english Search modes - Boolean/Phrase | Interface - EBSCOhost Research Databases Search Screen - Advanced Search Database - CINAHL Plus with Full Text | 12,312 |
| S19 | S17 NOT S18 | Search modes - Boolean/Phrase | Interface - EBSCOhost Research Databases Search Screen - Advanced Search Database - CINAHL Plus with Full Text | 12,549 |
| S18 | (MH "Animals+") NOT (MH "Human") | Search modes - Boolean/Phrase | Interface - EBSCOhost Research Databases Search Screen - Advanced Search Database - CINAHL Plus with Full Text | 95,937 |
| S17 | S7 AND S16 | Search modes - Boolean/Phrase | Interface - EBSCOhost Research Databases Search Screen - Advanced Search Database - CINAHL Plus with Full Text | 12,572 |
| S16 | S8 OR S9 OR S10 OR S11 OR S12 OR S13 OR S14 OR S15 | Search modes - Boolean/Phrase | Interface - EBSCOhost Research Databases Search Screen - Advanced Search Database - CINAHL Plus with Full Text | 439,808 |
| S15 | TI ( (coproduc* or "co-produc*" or cocreat* or "co-creat*" or cocare or "co-care" or cocaring or "co-caring" or cocared or "co-cared" or cocommiss* or "co-commiss*" or "Co-decide" or "co-decision*" or "co-decided" or Codeci* or "Co-deliver*" or Codeliver* or "co-evaluat*" or Coevaluat* or "Co-implement*" or Coimplement* or "Co-construct*" or Coconstruct* or "Co-innovat*" or Coinnovat* or "Co-learn*" or Colearn*) ) OR AB ( (coproduc* or "co-produc*" or cocreat* or "co-creat*" or cocare or "co-care" or cocaring or "co-caring" or cocared or "co-cared" or cocommiss* or "co-commiss*" or "Co-decide" or "co-decision*" or "co-decided" or Codeci* or "Co-deliver*" or Codeliver* or "co-evaluat*" or Coevaluat* or "Co-implement*" or Coimplement* or "Co-construct*" or Coconstruct* or "Co-innovat*" or Coinnovat* or "Co-learn*" or Colearn*) ) | Search modes - Boolean/Phrase | Interface - EBSCOhost Research Databases Search Screen - Advanced Search Database - CINAHL Plus with Full Text | 3,890 |
| S14 | TI ( ((Patient* or user* or consumer* or stakeholder* or public* or client* or clinician* or provider* or caregiver* or communit* or inpatient* or outpatient* or people* or person* or individual*) N1 (centered or centred or centric or participa* or engag* or empower* or involv* or voice or view or perspective or experience* or advoca* or behav* or evaluat* or communicat* or collaborat* or opinion* or input* or consult* or partner* or develop* or design* or dialog*)) ) OR AB ( ((Patient* or user* or consumer* or stakeholder* or public* or client* or clinician* or provider* or caregiver* or communit* or inpatient* or outpatient* or people* or person* or individual*) N1 (centered or centred or centric or participa* or engag* or empower* or involv* or voice or view or perspective or experience* or advoca* or behav* or evaluat* or communicat* or collaborat* or opinion* or input* or consult* or partner* or develop* or design* or dialog*)) ) | Search modes - Boolean/Phrase | Interface - EBSCOhost Research Databases Search Screen - Advanced Search Database - CINAHL Plus with Full Text | 394,057 |
| S13 | TI (action N1 research) OR AB (action N1 research) | Search modes - Boolean/Phrase | Interface - EBSCOhost Research Databases Search Screen - Advanced Search Database - CINAHL Plus with Full Text | 5,919 |
| S12 | TI ( (design* N1 (particip* or "experience based" or "person* based")) ) OR AB ( (design* N1 (particip* or "experience based" or "person* based")) ) | Search modes - Boolean/Phrase | Interface - EBSCOhost Research Databases Search Screen - Advanced Search Database - CINAHL Plus with Full Text | 6,952 |
| S11 | TI ( (codesign* or "co-design*") ) OR AB ( (codesign* or "co-design*") ) | Search modes - Boolean/Phrase | Interface - EBSCOhost Research Databases Search Screen - Advanced Search Database - CINAHL Plus with Full Text | 1,537 |
| S10 | (MH "Patient Centered Care") | Search modes - Boolean/Phrase | Interface - EBSCOhost Research Databases Search Screen - Advanced Search Database - CINAHL Plus with Full Text | 34,999 |
| S9 | (MH "Consumer Participation") | Search modes - Boolean/Phrase | Interface - EBSCOhost Research Databases Search Screen - Advanced Search Database - CINAHL Plus with Full Text | 23,610 |
| S8 | (MH "Rehabilitation, Community-Based") | Search modes - Boolean/Phrase | Interface - EBSCOhost Research Databases Search Screen - Advanced Search Database - CINAHL Plus with Full Text | 1,158 |
| S7 | S1 OR S2 OR S3 OR S4 OR S5 OR S6 | Search modes - Boolean/Phrase | Interface - EBSCOhost Research Databases Search Screen - Advanced Search Database - CINAHL Plus with Full Text | 167,979 |
| S6 | TI ( ((cerebral or brain* or intracerebral or subarachnoid or "sub arachnoid" or intracranial or "intra cranial" or subdural or "sub dural" or cerebell* or vertebrobasil* or hemispher* or infratentorial or supratentorial or "middle cerebr*" or mca* or "anterior circulation") N3 (infarct* or haemorrhag* or hemorrhag* or ischemia* or ischaemia* or bleed* or thrombos* or thrombus* or embolism* or embolus or embolitic or occlus* or hypoxi*)) ) OR AB ( ((cerebral or brain* or intracerebral or subarachnoid or "sub arachnoid" or intracranial or "intra cranial" or subdural or "sub dural" or cerebell* or vertebrobasil* or hemispher* or infratentorial or supratentorial or "middle cerebr*" or mca* or "anterior circulation") N3 (infarct* or haemorrhag* or hemorrhag* or ischemia* or ischaemia* or bleed* or thrombos* or thrombus* or embolism* or embolus or embolitic or occlus* or hypoxi*)) ) | Search modes - Boolean/Phrase | Interface - EBSCOhost Research Databases Search Screen - Advanced Search Database - CINAHL Plus with Full Text | 33,656 |
| S5 | TI ( ((cerebrovasc* or "cerebr* vasc*") N3 (accident* or disorder* or disease* or event* or incident* or infarct*)) ) OR AB ( ((cerebrovasc* or "cerebr* vasc*") N3 (accident* or disorder* or disease* or event* or incident* or infarct*)) ) | Search modes - Boolean/Phrase | Interface - EBSCOhost Research Databases Search Screen - Advanced Search Database - CINAHL Plus with Full Text | 11,080 |
| S4 | TI ( (stroke* or poststroke* or "transient ischemic attack*") ) OR AB ( (stroke* or poststroke* or "transient ischemic attack*") ) | Search modes - Boolean/Phrase | Interface - EBSCOhost Research Databases Search Screen - Advanced Search Database - CINAHL Plus with Full Text | 114,242 |
| S3 | (MH "Stroke Patients") | Search modes - Boolean/Phrase | Interface - EBSCOhost Research Databases Search Screen - Advanced Search Database - CINAHL Plus with Full Text | 7,747 |
| S2 | (MH "Hypoxia-Ischemia, Brain") | Search modes - Boolean/Phrase | Interface - EBSCOhost Research Databases Search Screen - Advanced Search Database - CINAHL Plus with Full Text | 1,487 |
| S1 | (MH "Intracranial Embolism and Thrombosis") OR (MH "Intracranial Embolism") OR (MH "Intracranial Hemorrhage+") OR (MH "Stroke") OR (MH "Cerebral Infarction") OR (MH "Hemorrhagic Stroke") OR (MH "Stroke, Lacunar") OR (MH "Ischemic Stroke") OR (MH "Embolic Stroke") OR (MH "Hypoxia-Ischemia, Brain") OR (MH "Cerebrovascular Disorders") OR (MH "Intracranial Thrombosis") OR (MH "Sinus Thrombosis, Intracranial") OR (MH "CADASIL") | Search modes - Boolean/Phrase | Interface - EBSCOhost Research Databases Search Screen - Advanced Search Database - CINAHL Plus with Full Text | 104,757 |
|  |  |  |  |  |

Cochrane

ID Search Hits

#1 [mh ^"intracranial embolism and thrombosis"] or [mh ^"intracranial embolism"] or [mh "intracranial thrombosis"] or [mh "intracranial hemorrhages"] or [mh ^stroke] or [mh ^"brain infarction"] or [mh ^"brain stem infarctions"] or [mh ^"lateral medullary syndrome"] or [mh ^"cerebral infarction"] or [mh ^"cadasil"] or [mh ^"dementia, multi-infarct"] or [mh ^"infarction, anterior cerebral artery"] or [mh ^"infarction, middle cerebral artery"] or [mh ^"infarction, posterior cerebral artery"] 13685

#2 [mh ^"cerebrovascular disorders"] or [mh ^"hypoxia-ischemia, brain"] 1724

#3 [mh ^"hemorrhagic stroke"] or [mh ^"ischemic stroke"] or [mh ^"embolic stroke"] or [mh ^"thrombotic stroke"] or [mh ^"stroke, lacunar"] 470

#4 [mh ^"Stroke Rehabilitation"] 3014

#5 (stroke* or poststroke* or "transient ischemic attack*"):ti,ab,kw 66402

#6 ((cerebrovasc* or "cerebr* vasc*") NEAR/3 (accident* or disorder* or disease* or event* or incident* or infarct*)):ti,ab,kw 21240

#7 ((cerebral or brain* or intracerebral or subarachnoid or "sub arachnoid" or intracranial or "intra cranial" or subdural or "sub dural" or cerebell* or vertebrobasil* or hemispher* or infratentorial or supratentorial or "middle cerebr*" or mca* or "anterior circulation") NEAR/3 (infarct* or haemorrhag* or hemorrhag* or ischemia* or ischaemia* or bleed* or thrombos* or thrombus* or embolism* or embolus or embolitic or occlus* or hypoxi*)):ti,ab,kw 21530

#8 #1 OR #2 OR #3 OR #4 OR #5 OR #6 OR #7 82323

#9 [mh ^"Community-Based Participatory Research"] 278

#10 [mh ^"community participation"] or [mh ^"patient participation"] 1836

#11 [mh ^"Patient-Centered Care"] 701

#12 (codesign* or "co-design*"):ti,ab,kw 345

#13 (design* NEAR/1 (particip* or "experience based" or "person* based")):ti,ab,kw 2509

#14 (action NEAR/1 research):ti,ab,kw 918

#15 ((Patient* or user* or consumer* or stakeholder* or public* or client* or clinician* or provider* or caregiver* or communit* or inpatient* or outpatient* or people* or person* or individual*) NEAR/1 (centered or centred or centric or participa* or engag* or empower* or involv* or voice or view or perspective or experience* or advoca* or behav* or evaluat* or communicat* or collaborat* or opinion* or input* or consult* or partner* or develop* or design* or dialog*)):ti,ab,kw 79385

#16 (coproduc* or "co-produc*" or cocreat* or "co-creat*" or cocare or "co-care" or cocaring or "co-caring" or cocared or "co-cared" or cocommiss* or "co-commiss*" or "Co-decide" or "co-decision*" or "co-decided" or Codeci* or "Co-deliver*" or Codeliver* or "co-evaluat*" or Coevaluat* or "Co-implement*" or Coimplement* or "Co-construct*" or Coconstruct* or "Co-innovat*" or Coinnovat* or "Co-learn*" or Colearn*):ti,ab,kw 375

#17 #9 OR #10 OR #11 OR #12 OR #13 OR #14 OR #15 OR #16 82820

#18 #8 AND #17 4568

#19 [mh Animals] not [mh Humans] 13

#20 #18 NOT #19 4568

Scopus

( ( TITLE-ABS-KEY ( ( stroke* OR poststroke* OR "transient ischemic attack*" ) ) OR TITLE-ABS-KEY ( ( ( cerebrovasc* OR "cerebr* vasc*" ) W/3 ( accident* OR disorder* OR disease* OR event* OR incident* OR infarct* ) ) ) OR TITLE-ABS-KEY ( ( ( cerebral OR brain* OR intracerebral OR subarachnoid OR "sub arachnoid" OR intracranial OR "intra cranial" OR subdural OR "sub dural" OR cerebell* OR vertebrobasil* OR hemispher* OR infratentorial OR supratentorial OR "middle cerebr*" OR mca* OR "anterior circulation" ) W/3 ( infarct* OR haemorrhag* OR hemorrhag* OR ischemia* OR ischaemia* OR bleed* OR thrombos* OR thrombus* OR embolism* OR embolus OR embolitic OR occlus* OR hypoxi* ) ) ) ) ) AND ( ( TITLE-ABS-KEY ( ( codesign* OR "co-design*" ) ) OR TITLE-ABS-KEY ( ( design* W/1 ( particip* OR "experience based" OR "person* based" ) ) ) OR TITLE-ABS-KEY ( ( action W/1 research ) ) OR TITLE-ABS-KEY ( ( ( patient* OR user* OR consumer* OR stakeholder* OR public* OR client* OR clinician* OR provider* OR caregiver* OR communit* OR inpatient* OR outpatient* OR people* OR person* OR individual* ) W/1 ( centered OR centred OR centric OR participa* OR engag* OR empower* OR involv* OR voice OR view OR perspective OR experience* OR advoca* OR behav* OR evaluat* OR communicat* OR collaborat* OR opinion* OR input* OR consult* OR partner* OR develop* OR design* OR dialog* ) ) ) OR TITLE-ABS-KEY ( ( coproduc* OR "co-produc*" OR cocreat* OR "co-creat*" OR cocare OR "co-care" OR cocaring OR "co-caring" OR cocared OR "co-cared" OR cocommiss* OR "co-commiss*" OR "Co-decide" OR "co-decision*" OR "co-decided" OR codeci* OR "Co-deliver*" OR codeliver* OR "co-evaluat*" OR coevaluat* OR "Co-implement*" OR coimplement* OR "Co-construct*" OR coconstruct* OR "Co-innovat*" OR coinnovat* OR "Co-learn*" OR colearn* ) ) ) ) AND ( LIMIT-TO ( LANGUAGE , "English" ) )

Global Index Medicus

tw:(tw:(tw:((tw:(stroke* OR poststroke* OR "transient ischemic attack*" OR "cerebrovasc* accident*" OR "cerebrovasc* disorder*" OR "cerebrovasc* disease*" OR "cerebrovasc* event*" OR "cerebrovasc* incident*" OR "cerebrovasc* infarct*" OR "cerebr* vasc* accident*" OR "cerebr* vasc* disorder*" OR "cerebr* vasc* disease*" OR "cerebr* vasc* event*" OR "cerebr* vasc* incident*" OR "cerebr* vasc* infarct*" OR "intracranial embolism" OR "intracranial thrombosis" OR "intracranial hemorrhage*" OR "intracranial infarction*" OR "cerebral infarct*" OR "cerebral haemorrhag*" OR "cerebral hemorrhag*" OR "cerebral ischemia*" OR "cerebral ischaemia*" OR "cerebral bleed*" OR "cerebral thrombos*" OR "cerebral thrombus*" OR "cerebral embolism*" OR "cerebral embolus" OR "cerebral embolitic" OR "cerebral occlus*" OR "cerebral hypoxi*" OR "brain* infarct*" OR "brain* haemorrhag*" OR "brain* hemorrhag*" OR "brain* ischemia*" OR "brain* ischaemia*" OR "brain* bleed*" OR "brain* thrombos*" OR "brain* thrombus*" OR "brain* embolism*" OR "brain* embolus" OR "brain* embolitic" OR "brain* occlus*" OR "brain* hypoxi*" OR "intracerebral infarct*" OR "intracerebral haemorrhag*" OR "intracerebral hemorrhag*" OR "intracerebral ischemia*" OR "intracerebral ischaemia*" OR "intracerebral bleed*" OR "intracerebral* thrombos*" OR "intracerebral thrombus*" OR "intracerebral embolism*" OR "intracerebral embolus" OR "intracerebral embolitic" OR "intracerebral occlus*" OR "intracerebral hypoxi*" OR "intra cranial infarct*" OR "intra cranial haemorrhag*" OR "intra cranial hemorrhag*" OR "intra cranial ischemia*" OR "intra cranial ischaemia*" OR "intra cranial bleed*" OR "intra cranial thrombos*" OR "intra cranial thrombus*" OR "intra cranial embolism*" OR "intra cranial embolus" OR "intra cranial embolitic" OR "intra cranial occlus*" OR "intra cranial hypoxi*")) AND (tw:(codesign* OR "co-design*" OR "particip* design*" OR "experience based design*" OR "person* based design*" OR "action research" OR coproduc* OR "co-produc*" OR cocreat* OR "co-creat*" OR cocare OR "co-care" OR cocaring OR "co-caring" OR cocared OR "co-cared" OR cocommiss* OR "co-commiss*" OR "Co-decide" OR "co-decision*" OR "co-decided" OR codeci* OR "Co-deliver*" OR codeliver* OR "co-evaluat*" OR coevaluat* OR "Co-implement*" OR coimplement* OR "Co-construct*" OR coconstruct* OR "Co-innovat*" OR coinnovat* OR "Co-learn*" OR colearn* OR "patient* center*" OR "patient* centr*" OR "Patient participa*" OR "patient engag*" OR "patient* empower*" OR "patient* experience" OR "patient* voice" OR "patient* view" OR "patient* perspective*" OR "patient* experience*" OR "patient* advoca*" OR "patient* collaborat*" OR "patient* opinion*" OR "patient* input*" OR "patient* consult*" OR "patient* dialog*" OR "user* center*" OR "user* centr*" OR "user participa*" OR "user engag*" OR "user* empower*" OR "user* experience" OR "user* voice" OR "user* view" OR "user* perspective*" OR "user* experience*" OR "user* advoca*" OR "user* collaborat*" OR "user* opinion*" OR "user* input*" OR "user* consult*" OR "user* dialog*" OR "consumer* center*" OR "consumer* centr*" OR "consumer* participa*" OR "consumer* engag*" OR "consumer* empower*" OR "consumer* experience" OR "consumer* voice" OR "consumer* view" OR "consumer* perspective*" OR "consumer* experience*" OR "consumer* advoca*" OR "consumer* collaborat*" OR "consumer* opinion*" OR "consumer* input*" OR "consumer* consult*" OR "consumer* dialog*"))))) AND ( la:("en"))
