# Supplementary material for: Co-design for stroke intervention development: Results of a scoping review
Source: PLoS One. 2024 Feb 14;19(2):e0297162. doi: 10.1371/journal.pone.0297162 (PMC10866508; doi:10.1371/journal.pone.0297162)
Supplement: S1 Table — (DOCX) [file pone.0297162.s002.docx]

| **Authors** | **Title** | **Year Published** | **Journal/Gray literature source** |
| --- | --- | --- | --- |
| Abdul A., Aznida F.; Mohd N., Nor A.; Ali, M. F.; Abd A., Noor A.; Sulong, S.; Aljunid, S. M. | The integrated care pathway for post stroke patients (iCaPPS): a shared care approach between stakeholders in areas with limited access to specialist stroke care services | 2017 | BMC health services research |
| Ahmed, R.; Goodall, S.; Bagguley, J. | LINCOLNSHIRE STROKE SERVICE UK: A SERVICE FIT FOR OUR OWN FAMILIES | 2022 | European stroke journal |
| Alhusayni, A.; Cowey, E.; Dybus, A.; Paul, L. | Improving acceptability and suitability of web-based physiotherapy for people undergoing stroke rehabilitation and their carers using co-production methods...Chartered Society of Physiotherapy Conference & Trade Exhibition, Physiotherapy UK 2019, Nov 1, 2019-Nov 2, 2019, United Kingdom | 2020 | Physiotherapy |
| Aljaroodi, H. M.; Adam, M. T. P.; Chiong, R.; Cornforth, D. J.; Minichiello, M. | Empathic avatars in stroke rehabilitation: A co-designed mhealth artifact for stroke survivors | 2017 | International Conference on Design Science Research in Information System and Technology |
| Allison, R.; Evans, P. H.; Kilbride, C.; Campbell, J. L. | Secondary prevention of stroke: using the experiences of patients and carers to inform the development of an educational resource | 2008 | Family practice |
| Alves, T.; Carvalho, H.; Simoes L., D. | Winning compensations: Adaptable gaming approach for upper limb rehabilitation sessions based on compensatory movements | 2020 | Journal of biomedical informatics |
| Amaya, A., Woolf, C., Devane, N., Galliers, J., Talbot., Wilson, S., Marshall, J. | Receiving aphasia intervention in a virtual environment: the participants’ perspective | 2018 | Aphasiology |
| Anderson, J. A.; Godwin, K. M.; Saleem, J. J.; Russell, S.; Robinson, J. J.; Kimmel, B. | Accessibility, usability, and usefulness of a Web-based clinical decision support tool to enhance provider-patient communication around Self-management TO Prevent (STOP) Stroke | 2014 | Health informatics journal |
| Anemaat, L.; Palmer, V. J.; Copland, D. A.; Mainstone, K.; Druery, K.; Druery, J.; Aisthorpe, B.; Binge, G.; Mainstone, P.; Wallace, S. J. | Using experience-based codesign to coproduce aphasia rehabilitation services: study protocol | 2021 | BMJ open |
| Armstrong, E.; Colegate, K.; Papertalk, L.; Woods, D.; Thompson, S.; Katzenellenbogen, J.; Ciccone, N.; Godecke, E.; Hersh, D.; McAllister, M.; Coffin, J. | Yarning circles providing support for Aboriginal Australians after acquired brain injury | 2022 | International Journal of Stroke |
| Auger, L.; Allegue, D. R.; Morales, E.; Thomas, A.; Filiatrault, J.; Vachon, B.; Rochette, A. | Co-designing a Program to Improve Post-stroke Sexual Rehabilitation: The Promise of Innovative Methods | 2022 | Frontiers in rehabilitation sciences |
| Badder, J.; Butler, L.; Christoff, K.; Oldershaw, B. | Ensuring the message is received: Adopting a patient oriented discharge summary approach to discharge | 2018 | International Journal of Stroke |
| Bagot, K. L.; Cadilhac, D. A.; Kim, J.; Vu, M.; Savage, M.; Bolitho, L.; Howlett, G.; Rabl, J.; Dewey, H. M.; Hand, P. J.; Denisenko, S.; Donnan, G. A.; Bladin, C. F. | Transitioning from a single-site pilot project to a state-wide regional telehealth service: The experience from the Victorian Stroke Telemedicine programme | 2017 | Journal of Telemedicine & Telecare |
| Bajuaifer, S.; Grey, M. J.; Hancock, N.; Pomeroy, V. M. | User perspectives on the design and setup of lower limb mirror therapy equipment after stroke: a technical report | 2021 | Physiotherapy |
| Balaam, M.; Egglestone, S. R.; Fitzpatrick, G.; Rodden, T.; Hughes, A. M.; Wilkinson, A.; Nind, T.; Axelrod, L.; Harris, E.; Ricketts, I.; Mawson, S.; Burridge, J. | Motivating mobility: Designing for lived motivation in stroke rehabilitation | 2011 | Conference on Human Factors in Computing Systems - Proceedings |
| Balatsoukas, P.; Porat, T.; Sassoon, I.; Essers, K.; Kokciyan, N.; Chapman, M.; Drake, A.; Modgil, S.; Ashworth, M.; Sklar, E.; Parsons, S.; Curcin, V. | User Involvement in the Design of a Data-Driven Self-Management Decision Support Tool for Stroke Survivors | 2019 | IEEE EUROCON 2019 -18th International Conference on Smart Technologies |
| Baranyi, R.; Czech, P.; Hofstatter, S.; Aigner, C.; Grechenig, T. | Analysis, Design, and Prototypical Implementation of a Serious Game Reha@Stroke to Support Rehabilitation of Stroke Patients with the Help of a Mobile Phone | 2020 | IEEE Transactions on Games |
| Barnden, R.; Cadilhac, D.; Lannin, N.; Kneebone, I.; Hersh, D.; Godecke, E.; Stolwyk, R.; Purvis, T.; Howard, K.; Nicks, R.; Farquhar, M.; Gleeson, S.; Gore, C.; Andrew, N. E. | Development and field testing of a standardised goal setting package for patientcentered discharge care planning in stroke | 2018 | International Journal of Stroke |
| Belson, S.; Robinson-Edwards, N. | Creating an educational space to raise awareness of post stroke spasticity treatment options through a stroke support organization and healthcare company collaboration | 2018 | International Journal of Stroke |
| Bharmal, N.; Mittman, B. S.; Shah, R.; Sarkisian, C. | Cultural tailoring of stroke risk factor reduction and walking promotion community intervention for South Asian older adult immigrants | 2014 | Journal of general internal medicine |
| Black, C.; Liu, D.; Petrash, H.; Warga, G. | The Knee Nook | 2008 | Topics in stroke rehabilitation |
| Blanco, T.; Casas, R.; Marco, A.; MartÃ­nez, I. | Micro ad-hoc Health Social Networks (uHSN). Design and evaluation of a social-based solution for patient support | 2019 | Journal of biomedical informatics |
| Bosch, J.; DePaul, V. G.; Oczkowski, W. J.; Wishart, L. R.; Draaistra, H.; O'Donnell, M.; Hart, R. | What about the other 12 hours? Development and pilot testing of a novel program to increase walking-related practice during stroke rehabilitation | 2014 | Stroke |
| Bowman, D. A.; Jenkins, H. | There's an iBook for that: Contemporary technology helps meet stroke patients' health literacy needs | 2015 | International Journal of Stroke |
| Brandy, V.; Janssen, H.; Lackay, L.; Smallwood, R.; Usher, K.; Peake, R.; Hasnain, M. D.; Fu, V.; McNaughton, H.; Garcia-Esperon, C.; Spratt, N. J.; Levi, C. | Yarning up After Stroke: Phase I - Community led development of a self-management tool to empower First Nations People of Australia to determine their stroke recovery | 2021 | International Journal of Stroke |
| Brown, S. E.; Scobbie, L.; Worrall, L.; Mc Menamin, R.; Brady, M. C. | Access G-AP: development of an accessible goal setting and action planning resource for stroke survivors with aphasia | 2022 | Disability and rehabilitation |
| Bushnell, C. D.; Duncan, P. W.; Lycan, S. L.; Condon, C. N.; Pastva, A. M.; Lutz, B. J.; Halladay, J. R.; Cummings, D. M.; Arnan, M. K.; Jones, S. B.; et al., | A Person-Centered Approach to Poststroke Care: the COMprehensive Post-Acute Stroke Services Model | 2018 | Journal of the American Geriatrics Society |
| Cadilhac, D. A.; Busingye, D.; Li, J. C.; Andrew, N. E.; Kilkenny, M. F.; Thrift, A. G.; Thijs, V.; Hackett, M. L.; Kneebone, I.; Lannin, N. A.; et al., | Development of an electronic health message system to support recovery after stroke: inspiring virtual enabled resources following vascular events (iVERVE) | 2018 | Patient preference and adherence |
| Calder, A., Sole, G., Mulligan, H. | Co-Design of an Educational Resource with Female Partners of  Male Stroke Survivors to Support Physical Activity Participation | 2022 | International Journal of Environmental Research and Public Health |
| Cameron, T.; Chouliara, N.; Jones, F.; Lee, R.; Blake, M.; Stock, L.; Meads, T.; Fisher, R. | Creation of a co-designed interdisciplinary team (IDT) development programme to facilitate evidence-based rehabilitation of stroke survivors in the acute setting (concord project) | 2020 | International Journal of Stroke |
| Carragher, M.; O'Halloran, R.; Johnson, H.; Taylor, N.; Torabi, T.; Rose, M. | The communication gap between staff and people with aphasia: There's an app for that! | 2018 | Brain Impairment |
| Caunca, Michelle R.; Simonetto, Marialaura; Hartley, Gregory; Wright, Clinton B.; Czaja, Sara J. | Design and Usability Testing of the Stroke Caregiver Support System: A Mobile-Friendly Website to Reduce Stroke Caregiver Burden | 2020 | Rehabilitation nursing : the official journal of the Association of Rehabilitation Nurses |
| Cheung, D.; French, E.; Fleck, R.; Huffman, S.; Kelloway, L.; Linkewich, B.; Maebar-Waller, A.; Murphy, C.; Nugent, B.; Quant, S.; Sooley, D.; Traetto, M.; Willems, D. | A provincial collaboration to enhance stroke early supported discharge knowledge and resources | 2015 | International Journal of Stroke |
| Cheung, D.; Morley, M.; O'Neill, J.; Willems, J. | Dissemination of a stroke aboriginal resource guide for health service providers working with urban aboriginal peoples | 2012 | Stroke |
| Choo, P. L.; Zhang, Y. | Co-designing stroke rehabilitation units to promote physical activity after stroke | 2020 | International Journal of Stroke |
| Clarke, D.; Gombert, K.; Robert, G.; Harris, R.; McKevitt, C.; Honey, S.; Macdonald, A.; Cloud, G.; Jones, F. | Increasing social, cognitive and physical activity on acute stroke units using an experience-based codesign approach: the collaborative rehabilitation environments in acute stroke (create) study | 2019 | European stroke journal |
| Clarke, D.; Morton, S.; Fitzsimons, C.; Hall, J.; Corepal, R.; Lawton, R.; Gillian, M.; Birch, K.; Farrin, A.; Holloway, I.; Patel, A.; English, C.; Forster, A. | Co-producing a complex intervention to reduce sedentary behaviour after stroke: Challenges and solutions | 2019 | European stroke journal |
| Clarke, D.; Gombert-Waldron, K.; Honey, S.; Cloud, G.; Harris, R.; Macdonald, A.; McKevitt, C.; Robert, G.; Jones, F. | Co-designing organisational improvements and interventions to increase inpatient activity in four stroke units in England: a mixed-methods process evaluation using normalisation process theory | 2021 | BMJ open |
| Clatworthy, P.; Turton, A.; Angilley, J.; Palombi, A.; Hazelton, C.; Harding, S.; Cashell, A.; Bowen, A.; Rowe, F. | Hemianopia activity-based intervention: co-design of an occupational therapy intervention for visual field loss after stroke | 2021 | International Journal of Stroke |
| Corches, C. L,; McBride, A C.; Robles, M. C.; Rehman, N.; Bailey, S.; Oliver, A.; Skolarus, L. E. | Development, adaptation and scale-up of a community-wide, health behavior theory based stroke preparedness intervention | 2020 | American journal of health behavior |
| Cregg, A.; Grimshaw, S.; Hogan, A.; Shaw, E.; Ackerley, S. | Describing neuro rehabilitation on-line (NROL): 'cogs in motion' cognitive rehabilitation group | 2021 | International Journal of Stroke |
| Dancer, S. | Redesigning care for the nonhemorrhagic stroke patient | 1996 | The Journal of neuroscience nursing : journal of the American Association of Neuroscience Nurses |
| Davoody, N. | From chaos to clarity: Designing eHealth to support self-management in stroke care | 2022 | Dissertation Abstracts International Section A: Humanities and Social Sciences |
| Deloge, E.; Karna, M.; Guarna, F.; Cox, N.; Labranche, D.; Lattas, M.; Jomaa, C.; Minuk, J. | Creation of a Stroke Integrated Practice Unit | 2019 | International Journal of Stroke |
| Demers, M.; Mbiya, N.; Levin, M. F. | Industry and academia collaboration in the design of virtual reality applications for rehabilitation | 2017 | 2017 International Conference on Virtual Rehabilitation |
| Ding, E. Y.; Han, D.; Dickson, E. L.; DiMezza, D.; Scott, J.; Mohagheghian, F.; Peitzsch, A.; Saczynski, J.; Moonis, M.; Fitzgibbons, T. P.; et al., | Use of a smartwatch and app designed by stroke survivors for atrial fibrillation detection in older adults after stroke/transient ischemic event: preliminary findings from an ongoing randomized clinical trial | 2021 | Circulation |
| Donetto, S.; Jones, F.; Clarke, D. J.; Cloud, G. C.; Gombert-Waldron, K.; Harris, R.; Macdonald, A.; McKevitt, C.; Robert, G. | Exploring liminality in the co-design of rehabilitation environments: The case of one acute stroke unit | 2021 | Health and Place |
| Driver, S.; McShan, E.; Swank, C.; Grobe, K.; Calhoun, S.; Bailey, R.; Kramer, K. | Creating an appropriate adaptation of a healthy lifestyle intervention for people after stroke | 2020 | Brain injury |
| Drummond, A.; Ablewhite, J.; Condon, L.; das Nair, R.; Jones, A.; Jones, F.; Sprigg, N.; Thomas, S. | Developing a fatigue programme: Protocol for the Nottingham Fatigue After Stroke (NotFAST2) study | 2020 | The British journal of occupational therapy |
| Duval, J. S. | Playful health technology: A participatory, research through design approach to applications for wellness | 2023 | Dissertation Abstracts International: Section B: The Sciences and Engineering |
| Dyson, L.; Britten, M.; Mallett, A.; Sedman, P.; Sewards, I.; Hayden, G. | Pathway working in the Rotherham stroke rehabilitation team: Service development and lessons learnt | 2019 | International Journal of Stroke |
| El-Helou, Rebecca; Ryan, Brooke; Kneebone, Ian | Development of the "Kalmer" relaxation intervention: co-design with stroke survivors with aphasia | 2022 | Disability and rehabilitation |
| Eltringham, S. | Accessible information to support people with aphasia to make informed decisions about dysphagia management | 2021 | International Journal of Stroke |
| Engels, N.; Van Schaik, S.; Deijle, I.; Dahmen, R.; Brouwers, P.; Hilkens, P.; Garvelink, M.; The, R.; Van Uden-Kraan, C.; Van Der Wees, P.; Van Den Berg-Vos, R.; Prick, J. | Development of a patient decision aid regarding discharge planning for hospitalised patients with stroke | 2021 | European journal of neurology |
| English, C.; Attia, J. R.; Bernhardt, J.; Bonevski, B.; Burke, M.; Galloway, M.; Hankey, G. J.; Janssen, H.; Kuys, S.; Lindley, R. I.; et al., | Secondary Prevention of Stroke: study Protocol for a Telehealth-Delivered Physical Activity and Diet Pilot Randomized Trial (ENAbLE-Pilot) | 2021 | Cerebrovascular diseases (Basel, Switzerland) |
| Fairbrother, P.; McCloughan, L.; Adam, G.; Brand, R.; Brown, C.; Watson, M.; Cotter, N.; Mackellaig, J.; McKinstry, B. | Involving patients in clinical research: the Telescot Patient Panel | 2016 | Health expectations : an international journal of public participation in health care and health policy |
| Fini, N. A.; Ramage, E.; Bernhardt, J.; Bicknell, E.; Tzefronis, C.; Fink, P.; Godecke, E.; English, C.; Francis, J.; Wood, R.; Said, C. M. | Co-design of a Personalised Physical Activity Intervention for People After Stroke - Insights into Stakeholder Engagement | 2022 | International Journal of Stroke |
| Flynn, D.; Avery, L.; Hrisos, N.; Price, C. I.; Moore, S. | Development of an evidence-and theory-based physical activity/sedentary behavioural intervention for stroke survivors: Physical Activity Routines after Stroke (PARAS) | 2018 | International Journal of Stroke |
| Forat, T.; Liao, Z.; Curcin, V. | Engaging Stakeholders in the Design and Usability Evaluation of a Decision Aid to Improve Secondary Stroke Prevention..."Building Continents of Knowledge in Oceans of Data: The Future of Co-Created eHealth," EFMI, Medical Informatics Europe (MIE), April 24-26th, 2018, Gothenburg, Sweden | 2018 | Studies in Health Technology & Informatics |
| Forster, A.; Clarke, D.; Fitzsimons, C.; Birch, K.; Farrin, A.; Patel, A.; Lawton, R.; Mead, G.; Holloway, I.; English, C.; Carter, G. | The reducing sedentary behaviour after stroke study (recreate): Development and evaluation of an intervention to improve outcomes | 2018 | International Journal of Stroke |
| Freedman, D. E.; Behih, N.; Elmeligi, A.; Sahlas, D. | Keeping track of time: Emphasizing symptom onset-tohospital time in stroke care | 2019 | Canadian Journal of Neurological Sciences |
| Fritz, M. C.; Swierenga, S. J.; Freddolino, P. P.; Coursaris, C. K.; Woodward, A. T.; Reeves, M. J. | Lessons Learned in Developing a Patient-Centered Website to Support Stroke Patients and Caregivers During Transitions of Care | 2021 | Design, User Experience, and Usability: Design for Diversity, Well-being, and Social Development |
| Fusari, G.; Gibbs, E.; Hoskin, L.; Dickens, D.; Leis, M.; Taylor, E.; Jones, F.; Darzi, A. | Protocol for a feasibility study of OnTrack: a digital system for upper limb rehabilitation after stroke | 2020 | BMJ open |
| Gall, S.; Callisaya, M.; Schultz, M.; Sanderson, S.; Castley, H.; Walsh, K.; Peterson, G. | Cardiac rehabilitation for the secondary prevention of stroke (Caress): A pilot study of a co-designed program for people after stroke | 2020 | International Journal of Stroke |
| Georgiou, T.; Holland, S.; Van Der Linden, J.; Tetley, J.; Stockley, R. C.; Donaldson, G.; Garbutt, L.; Pinzone, O.; Grasselly, F.; Deleaye, K. | A blended user centred design study for wearable haptic gait rehabilitation following hemiparetic stroke | 2015 | 2015 9th International Conference on Pervasive Computing Technologies for Healthcare |
| Gesell, S. B.; Klein, K. P.; Halladay, J.; Bettger, J. P.; Freburger, J.; Cummings, D. M.; Lutz, B. J.; Coleman, S.; Bushnell, C.; Rosamond, W.; et al., | Methods guiding stakeholder engagement in planning a pragmatic study on changing stroke systems of care | 2017 | Journal of clinical and translational science |
| Gesell, S. B.; Coleman, S. W.; Mettam, L. H.; Johnson, A. M.; Sissine, M. E.; Duncan, P. W. | How engagement of a diverse set of stakeholders shaped the design, implementation, and dissemination of a multicenter pragmatic trial of stroke transitional care: The COMPASS study | 2020 | Journal of clinical and translational science |
| Gill, Colette; Lawless, Maura; O'Mara, Gerard; Macey, Chris; Dunning, Niamh; Dwyer, Ruth; Marrinan, Treasa | Supporting Stroke Survivors in Roscommon | 2017 | International Journal of Integrated Care (IJIC) |
| Gilmore, P.; Barry, J.; Blanchard, J.; Howson, S.; Korzycki, M. | Toolkit for return to work after stroke | 2012 | Stroke |
| Goldfinger, J. Z.; Kronish, I. M.; Fei, K.; Graciani, A.; Rosenfeld, P.; Lorig, K.; Horowitz, C. R. | Peer education for secondary stroke prevention in inner-city minorities: design and methods of the prevent recurrence of all inner-city strokes through education randomized controlled trial | 2012 | Contemporary clinical trials |
| Gombert, K.; Jones, F.; Cloud, G.; McKevitt, C.; Clarke, D.; Honey, S.; Harris, R.; Robert, G.; McDonald, A. | 'create' collaborative rehabilitation environments in acute stroke-an experience-based codesign approach (EBCD) to increasing activity of stroke patients in four hospitals in England | 2018 | European stroke journal |
| Gordon, C.; Ellis-Hill, C.; Dewar, B.; Watkins, C. | Knowing-in-action that centres humanising relationships on stroke units: An appreciative action research study | 2022 | Brain Impairment |
| Gregor, S. E. | Rhythm training for people with stroke: A tool to enhance neurorehabilitation | 2023 | Dissertation Abstracts International: Section B: The Sciences and Engineering |
| Gregory, S.; Matthew, J.; Dennis, L. J. S.; Scanlon, M.; Pipim-Addo, N. | Telehealth cognitive behavioral therapy to manage mood and emotions in the community | 2012 | International Journal of Stroke |
| Gretzinger, B. M.; Hirano, S. M.; Hebert, D.; Wang, R. | Using participatory action research to develop adaptive purses for women with chronic hemiparesis | 2015 | International Journal of Stroke |
| Groussard, P.; Pigot, H.; Giroux, S. | From conception to evaluation of mobile services for people with head injury: A participatory design perspective | 2018 | Neuropsychological rehabilitation |
| Groves, J.; Sanders, L.; Brock, K.; Borschmann, K.; Ilett, P.; Stone, L. | Innovative use of hospital space to increase physiotherapy interventions and enhance recovery of acute stroke patients | 2019 | International Journal of Stroke |
| Guidetti, S.; Gustavsson, M.; Fors, U.; Tham, K.; Andersson, M.; Ytterberg, C. | Development and evaluation of the effect and feasibility of a person-centred multidisciplinary intervention for rehabilitation after stroke | 2018 | International Journal of Stroke |
| Guneysu Ozgur, A.; Wessel, M. J.; Johal, W.; Sharma, K.; Zgür, A.; Vuadens, P.; Mondada, F.; Hummel, F. C.; Dillenbourg, P. | Iterative Design of an Upper Limb Rehabilitation Game with Tangible Robots | 2018 | 2018 13th ACM/IEEE International Conference on Human-Robot Interaction (HRI) |
| Gustafsson, L.; Bower, K.; Dobe, J.; Walder, K.; Lachman, R.; Kay, K. A. A.; Baggio, S.; Roberts, M.; Plastow, A. | The Right Information at the Right Time - Co-development of approaches to self-management support across the continuum of care | 2022 | International Journal of Stroke |
| Hall, J.; Clarke, D. J.; Forster, A. | Developing a theory and evidence-based intervention for carers of stroke survivors using an Intervention Mapping approach | 2018 | International Journal of Stroke |
| Hall, J.; Morton, S.; Hall, J.; Clarke, D. J.; Fitzsimons, C. F.; English, C.; Forster, A.; Mead, G. E.; Lawton, R. | A co-production approach guided by the behaviour change wheel to develop an intervention for reducing sedentary behaviour after stroke | 2020 | Pilot and feasibility studies |
| Hall, J. F.; Crocker, Thomas F.; Clarke, D. J.; Forster, A. | Supporting carers of stroke survivors to reduce carer burden: development of the Preparing is Caring intervention using Intervention Mapping | 2019 | BMC public health |
| Hamilton, H.; Clark, A.; McIntyre, H.; Reilly, S. | The implementation of a stroke patient hand held record in a remote and rural setting | 2009 | International Journal of Stroke |
| Harding, V.; Schmitt, J. | The surrey stroke recovery programme | 2018 | International Journal of Stroke |
| Holmes, R.; Sapardanis, N.; Truong, L. | Improving transitions in care with the stroke discharge checklist | 2019 | International Journal of Stroke |
| Holt, R.; Makower, S.; Jackson, A.; Culmer, P.; Levesley, M.; Richardson, R.; Cozens, A.; Williams, M. M.; Bhakta, B. | User involvement in developing rehabilitation robotic devices: An essential requirement | 2007 | 2007 IEEE 10th International Conference on Rehabilitation Robotics |
| Hopkins, E. M.; Hun, S.; Walkden, J.; Grigolo, S.; DeLuca, A.; Donnin, L. | Novel approach to procuring innovation in stroke rehabilitation | 2018 | International Journal of Stroke |
| Horowitz, C. R.; Fei, K.; Tuhrim, S.; Negron, R.; Kronish, I. | A peer-led intervention improves stroke survivors' blood pressure control | 2013 | Stroke |
| Horowitz, C.; Fei, K.; Kronish, I. M.; Tuhrim, S.; Negron, R. | A peer-led intervention improves stroke survivors' blood pressure control | 2013 | Journal of general internal medicine |
| Hu, X.; JonzÃ©n, K.; Karlsson, M.; Lindahl, O. A. | Assessments of a novel digital follow-up tool RehabkompassenÂ® to identify rehabilitation needs among stroke patients in an outpatient setting | 2022 | Digital health |
| Hughes, Anne K.; Woodward, Amanda T.; Fritz, Michele C.; Reeves, Mathew J. | Improving stroke transitions: Development and implementation of a social work case management intervention | 2018 | Social work in health care |
| Hughes, C.; Mariscal, T.; Baye, M.; Belay, G. J.; Hintze, A.; Padilla, A.; Warner, S.; Sera, M.; Gordon-Murer, C. | Development of an Upper Extremity Stroke Rehabilitation mHealth Application for sub-Saharan Africa: A Usability Study | 2019 | 2019 IST-Africa Week Conference (IST-Africa) |
| Hull, S.; Hartigan, N.; Kneebone, I. | Is bingo a psychological intervention? Developing a support group for stroke survivors and carers | 2007 | Clinical Psychology Forum |
| Hussein, H. M.; Droegemueller, C.; Xiong, P.; VanDyke, S.; Mettner, J.; X., Zong; Catha, G. | Hmong Cross-Cultural Adaptation of Stroke Educational Material | 2020 | WMJ : official publication of the State Medical Society of Wisconsin |
| Jarvis, K.; Bavikatte, G.; Branscombe, N.; Cook, J.; Haworth, J.; Lawrence, C.; Rose, A.; Thetford, C.; Stockley, R. | VIRTUAL ENGAGEMENT REHABILITATION ASSISTANT (VERA): FEASIBILITY, USABILITY AND ACCEPTABILITY OF A NOVEL DIGITAL HEALTH TECHNOLOGY IN COMPLEX REHABILITATION | 2022 | International Journal of Stroke |
| Jarvis, K.; Branscombe, N.; Cook, J.; Haworth, J.; Thetford, C.; Stockley, R. | An early implementation study of digital technology: A virtual engagement rehabilitation assistant (VERA) | 2021 | International Journal of Stroke |
| Jie, L.; Jamin, G.; Smit, K.; Beurskens, A.; Braun, S. | Design of the user interface for "Stappy", a sensor-feedback system to facilitate walking in people after stroke: a user-centred approach | 2020 | Disability and rehabilitation. Assistive technology |
| Johnson, V. L.; Apps, L.; Hadjiconstantinou, M.; Carey, M. E.; Kreit, E.; Mullis, R.; Mant, J.; Davies, M. J. | The development of a self-management intervention for stroke survivors - My Life After Stroke (MLAS) | 2022 | Disability and rehabilitation |
| Jones, F.; Gombert, K.; Clarke, D.; Honey, S.; Cloud, G.; Harris, R.; McKevitt, C.; Robert, G.; MacDonald, A. | CREATE 'Collaborative Rehabilitation Environments in Acute Stroke'-an experience-based co-design approach (EBCD) to improving activity experiences of stroke patients in 4 hospitals in England | 2019 | International Journal of Stroke |
| Jones, F.; Honey, S.; Gombert, K.; Cloud, G.; Harris, R.; Macdonald, A.; McKevitt, C.; Robert, G.; Clarke, D. J. | Collaborative Rehabilitation Environments in AcuTe strokE (CREATE)-experiences of co-designing changes in 2 stroke units | 2018 | International Journal of Stroke |
| Jones, F.; Gombert, K.; Honey, S.; Cloud, G.; Harris, R.; Macdonald, A.; McKevitt, C.; Robert, Glenn; Clarke, D. | Addressing inactivity after stroke: The Collaborative Rehabilitation in Acute Stroke (CREATE) study | 2021 | International journal of stroke : official journal of the International Stroke Society |
| Jones, F.; Gombert-Waldron, K.; Honey, S.; Cloud, G.; Harris, R.; Macdonald, A.; McKevitt, C.; Robert, G.; Clarke, D. | Using co-production to increase activity in acute stroke units: the CREATE mixed-methods study | 2020 | Health Services and Delivery Research |
| Jones, N. | Breakfast interventions in stroke rehabilitation (BISTRO) | 2021 | International Journal of Stroke |
| Kelliher, A.; Gibson, A.; Bottelsen, E.; Coe, E. | Designing modular rehabilitation objects for interactive therapy in the home | 2019 | Proceedings of the Thirteenth International Conference on Tangible, Embedded, and Embodied Interaction |
| Kerr, A.; Grealy, Madeleine A.; Kuschmann, A.; Rutherford, R.; Rowe, P. | A Co-creation Centre for Accessible Rehabilitation Technology | 2021 | Frontiers in rehabilitation sciences |
| Kilbride, C.; Nowicky, A.; Scott, D.; Warland, A.; Athanasiou, D.; Buxarrais, G. S. | GameBall: the development of a novel platform to provide enjoyable and affordable hand and arm rehabilitation following stroke | 2019 | Physiotherapy (United Kingdom) |
| Kilbride, C.; Warland, A.; Norris, M.; Ryan, J.; Anokye, N.; Scott, D.; Butcher, T.; Singla Buxarrais, G.; Athanasiou, D.; Nowicky, A. | RHOMBUS: Rehabilitation via HOMe Based gaming exercise for the Upper limb post-stroke | 2018 | International Journal of Stroke |
| Kilbride, C.; Scott, D. J. M.; Butcher, T.; Norris, M.; Ryan, J. M.; Anokye, N.; Warland, A.; Baker, K.; Athanasiou, D. A.; Singla-Buxarrais, G.; Nowicky, A. | Rehabilitation via home based gaming exercise for the upper-limb post stroke (rhombus): Protocol of an intervention feasibility trial | 2018 | BMJ Open |
| Kim, E. S.; Laird, L.; Wilson, C.; Bieg, T.; Mildner, P.; Möller, S.; Schatz, R.; Schwarz, S.; Spang, R.; Voigt-Antons, J. N.; Rochon, E. | Implementation and effects of an information technology-based intervention to support speech and language therapy among stroke patients with aphasia: Protocol for a virtual randomized controlled trial | 2021 | JMIR research protocols |
| Kingsley, C. M.; Hunter, L. | Co-design outcomes from sharing innovative practices and processes | 2011 | International Journal of Stroke |
| Kjork, E.; Stibrant Sunnerhagen, K.; Lundgren Nilsson, A.; Carlsson, G. | Evaluation of strokehealth, a digital tool to support follow-up after stroke | 2021 | European Stroke Journal |
| Kjork, E. K.; Sunnerhagen, K. S.; Lundgren-Nilsson, A.; Andersson, A. K.; Carlsson, G. | Development of a Digital Tool for People With a Long-Term Condition Using Stroke as a Case Example: Participatory Design Approach | 2022 | JMIR Human Factors |
| Krainer, D.; Wohofsky, L.; Schubert, P. | Design Requirements for a (Tele-) Rehabilitation Platform: Results from a Participatory Process | 2022 | Studies in Health Technology and Informatics |
| Krieger, T.; Boumans, N.; Feron, F.; Dorant, E. | The development of implementation management instruments for a new complex stroke caregiver intervention based on systematic stakeholder and risk analyses | 2020 | Scandinavian Journal of Caring sciences |
| Lam, S. K. Y.; Chau, J. P. C.; Lo, S. H. S.; Lau, A. Y. L.; Lee, V. W. Y.; Choi, K. C.; Mok, V. C. T.; Shum, E. W. C.; Hung, S. S.; Siow, E. K. C.; Ching, J. Y. L.; Yeung, J. H. M.; Li, S. H. | Development of an online multidisciplinary self-management programme for community-dwelling stroke survivors and caregivers | 2018 | International Journal of Stroke |
| Langford, T., Fleming, V., Upton, E., Doogan, C., Leff, A., Romano, D. M. | Design Innovation for Engaging and Accessible Digital Aphasia  Therapies: Framework Analysis of the iReadMore App Co-Design  Process | 2022 | JMIR Neurotechnology |
| Lawrence, M.; Davis, B.; De Amicis, L. | Heads: up mixed methods development study: working with stakeholders to adapt a mindfulness based stress reduction course for stroke survivors with mood disorder | 2022 | European stroke journal |
| Lee, K.; Fisher, K.; Markle-Reid, M.; Britt, E.; Dayler, D.; Hajas, K.; Koetsier, B.; Mahony, R.; Pagliuso, S.; Prescott, J.; Mayhew, L.; Walker, K. | Involving older adult stroke survivors and healthcare providers in designing and evaluating a patient-centred website to improve transitional care | 2021 | International Journal of Stroke |
| Lee, Min Hun | Interactive hybrid intelligence systems for human-AI/robot collaboration: Improving the practices of physical stroke rehabilitation | 2022 | Dissertation Abstracts International: Section B: The Sciences and Engineering |
| Lievesley, M.; Powell, R.; Carey, D.; Hulme, S.; O'Malley, L.; Westoby, W.; Zadik, J.; Bowen, A.; Brocklehurst, O.; Smith, C. J. | Co-designing for behaviour change: The development of a theory-informed oral-care intervention for stroke survivors | 2022 | Design for health (Abingdon, England) |
| Lin, P. J.; Huang, Y. H.; Hung, S. | Virtual Reality Training System for Rehabilitation of Patients with Stroke Based on Compensatory Movement Detection | 2020 | 2020 IEEE 2nd Eurasia Conference on Biomedical Engineering, Healthcare and Sustainability |
| Lindblom, Sebastian | Understanding the links: The exploration of care transitions between hospital and continued rehabilitation in the home after stroke | 2021 | Dissertation Abstracts International: Section B: The Sciences and Engineering |
| Lindblom, S.; Flink, M.; Elf, M.; Laska, A. C.; von Koch, L.; Ytterberg, C. | The manifestation of participation within a co-design process involving patients, significant others and health-care professionals | 2021 | Health expectations : an international journal of public participation in health care and health policy |
| Lobo, E. H.; Kensing, F.; Frolich, A.; Rasmussen, L. J.; Livingston, O. M.; Islam, S. M. S.; Grundy, J.; Abdelrazek, M. | mHealth intervention for carers of individuals with a history of stroke: Heuristic evaluation and user perspectives | 2022 | Digital health |
| Lund, A.; Michelet, M.; Kjeken, I.; Bruun Wyller, T.; Sveen, U. | Development of a person-centred lifestyle intervention for older adults following a stroke or transient ischaemic attack | 2012 | Scandinavian journal of occupational therapy |
| Lynch, E.; Cadilhac, D.; McGrath, A.; Harvey, G. | Co-creation of video information stories to address information needs of carers of stroke survivors | 2019 | International Journal of Stroke |
| Ma, M. Y.; Wu, F. G.; Chang, R. H. | A new design approach of user-centered design on a personal assistive bathing device for hemiplegia | 2007 | Disability and rehabilitation |
| Maddahi, A.; Bani Hani, J.; Asgari, A.; Nassiri, A. M.; Choukou, M. A. | Therapists' perspectives on a new portable hand telerehabilitation platform for home-based personalized treatment of stroke patients | 2021 | European review for medical and pharmacological sciences |
| Magnusson, C.; Caltenco, H. A.; McGookin, D.; Kytö, M.; Hjaltadóttir, I.; Hafsteinsdóttir, T. B.; Jónsdóttir, H.; Bjartmarz, I. | Tangible interaction for stroke survivors: Design recommendations | 2017 | Proceedings of the Eleventh International Conference on Tangible, Embedded, and Embodied Interaction |
| Magnusson, C.; Rassmus-GrÃ¶hn, K.; Rydeman, B.; Caltenco, H. | Walk after stroke - Initial development of a step counting game for stroke survivors | 2018 | Proceedings of the 20th International Conference on Human-Computer Interaction with Mobile Devices and Services Adjunct |
| Markle-Reid, M.; Fisher, K.; Walker, K.; Britt, E.; Dayler, D.; Hajas, K.; Koetsier, B.; Mahony, R.; Pagliuso, S.; Prescott, J. | WEBSITE FOR SUPPORTING SYSTEMNAVIGATION FOR OLDER ADULTS WITH STROKE AND MULTIMORBIDITY AND THEIR CAREGIVERS TRANSITIONING FROM HOSPITAL TO HOME: FEASIBILITY AND USABILITY STUDY | 2022 | International Journal of Stroke |
| Masterson-Algar, P.; Williams, S.; Burton, C. R.; Arthur, C. A.; Hoare, Z.; Morrison, V.; Radford, K.; Seddon, D.; Elghenzai, S. | Getting back to life after stroke: co-designing a peer-led coaching intervention to enable stroke survivors to rebuild a meaningful life after stroke | 2020 | Disability and rehabilitation |
| Mawson, S.; Nasr, N.; Parker, J.; Zheng, H.; Davies, R.; Mountain, G. | Developing a personalised self-management system for post stroke rehabilitation; utilising a user-centred design methodology | 2014 | Disability and rehabilitation. Assistive technology |
| McCue, P.; Shaw, L.; Del Din, S.; Hunter, H.; Lord, S.; Price, C. I. M.; Rodgers, H.; Rochester, L.; Moore, S. A. | Acceptability and deliverability of an auditory rhythmical cueing (ARC) training programme for use at home and outdoors to improve gait and physical activity post-stroke | 2022 | Archives of physiotherapy |
| McGowan, S.; Czerenkowski, J.; Hill, K. | Pwle reflections on impact: Stroke foundation Australia | 2021 | International Journal of Stroke |
| McGowan, S.; Czerenkowski, J.; O'Malley, A.; David, K.; Chamberlain, S. | Meeting information needs: Stroke Foundation's Young Stroke Project | 2022 | International Journal of Stroke |
| McKellar, J.; MacKay, D.; Mackie, J. | Peers fostering hope | 2012 | Stroke |
| Micallef, N.; Baillie, L.; Uzor, S. | Time to exercise! An aide-memoire stroke app for post-stroke arm rehabilitation | 2016 | Proceedings of the 18th International Conference on Human-Computer Interaction with Mobile Devices and Services |
| Moineau, B.; Myers, M.; Ali, S. S.; Popovic, M. R.; Hitzig, S. L. | End-user and clinician perspectives on the viability of wearable functional electrical stimulation garments after stroke and spinal cord injury | 2021 | Disability and rehabilitation. Assistive technology |
| Morris, Jacqui H.; Irvine, Linda A.; Dombrowski, Stephan U.; McCormack, Brendan; Van Wijck, Frederike; Lawrence, Maggie | We Walk: a person-centred, dyadic behaviour change intervention to promote physical activity through outdoor walking after stroke-an intervention development study | 2022 | BMJ open |
| Morris, R.; Falck, M.; Miles, T.; Wilcox, J.; Fisher-Hicks, S. | Rebuilding your life after stroke: Positive steps to well-being - a self-management book | 2017 | International Journal of Stroke |
| Morrow, J.; Chapman, J.; Badder, J.; Stevenson, G.; Butler, L.; Shea, B.; Smiley, C.; Steinmetz, H.; Honsberger, S.; Rodgers, T.; Reshwen, L.; Feore, C. | Successfully navigating to patient centered post stroke and post tia driving resources | 2018 | International Journal of Stroke |
| Nasr, Nasrin; Leon, Beatriz; Mountain, Gail; Nijenhuis, Sharon M.; Prange, Gerdienke; Sale, Patrizio; Amirabdollahian, Farshid | The experience of living with stroke and using technology: opportunities to engage and co-design with end users | 2016 | Disability and rehabilitation. Assistive technology |
| Naylor, R.; Wright, L.; Lalor, E. | My stroke care plan | 2012 | International Journal of Stroke |
| Neves, S.; Macdonald, A.; Poole, M.; Harrison Dening, K. | Participatory Co-design: Approaches to Enable People Living with Challenging Health Conditions to Participate in Design Research | 2021 | Springer Series in Design and Innovation |
| New, P.; Alpan, A.; Carrillo, F.; Sterling, L.; McCarthy, C.; Wise, L.; Skues, J.; Scheinberg, A. | Phase zero study for programming a sociable humanoid robot to increase activity in stroke rehabilitation inpatients | 2019 | International Journal of Stroke |
| Ng, E.; Wong, K. | Improving discharge transitions in inpatient stroke rehab with patient partner | 2019 | International Journal of Stroke |
| Ng, E.; Wong, K. | Community transitions in outpatient stroke rehab: Co-designed with persons with stroke, caregiver, and community partners | 2019 | International Journal of Stroke |
| O'Keefe, S.; Stanley, M.; Sansonetti, D.; Schneider, E. J.; Kras, M.; Morarty, J.; Lannin, N. A. | Designing an intervention process that embeds work-focussed interventions within inpatient rehabilitation: An intervention mapping approach | 2021 | Australian occupational therapy journal |
| Olafsdottir, Steinunn A.; Jonsdottir, Helga; Magnusson, Charlotte; Caltenco, Hector; Kyto, Mikko; Maye, Laura; McGookin, David; Bjartmarz, Ingibjorg; Arnadottir, Solveig Asa; Hjaltadottir, Ingibjorg; Hafsteinsdottir, Thora B. | Developing ActivABLES for community-dwelling stroke survivors using the Medical Research Council framework for complex interventions | 2020 | BMC health services research |
| O'Malley, A.; Browning, B.; Ridley, B.; Czerenkowski, J.; David, K.; Radulovic, M.; Browning, N.; Giacomin, P.; Burns, P.; Chamberlain, S.; Weaver, S.; Bowden, S.; Arfaras, T. | Stroke Foundation's Young Stroke Project: codesigning change | 2022 | International Journal of Stroke |
| Ortiz-Fernandez, L.; Alava-Menica, A.; Marti-Carrera, J.; Garcia-Fernandez, R.; Orcajo-Lago, J.; Arana-Arri, E.; Escobal-Gonzalo, J. | Patients as co-designers of the STARR system prototype | 2019 | Proceedings of the 13th EAI International Conference on Pervasive Computing Technologies for Healthcare |
| Osborne, Candice L.; Juengst, Shannon B.; Smith, Emily E. | Identifying user-centered content, design, and features for mobile health apps to support long-term assessment, behavioral intervention, and transitions of care in neurological rehabilitation: An exploratory study | 2021 | British Journal of Occupational Therapy |
| Palalon, H.; Simpson, J.; Arulvarathan, C.; Cayley, A.; Quraishi, F.; Sultan-Khan, L. | Standardizing documentation of interprofessional patient education in stroke inpatient care | 2018 | International Journal of Stroke |
| Peake, R.; Dieckmann, M. A.; Miller, J. I.; Stuhlmiller, C. | "Written by the mob for the mob": Collaborating with aboriginal communities to develop a culturally appropriate and relevant stroke education booklet | 2014 | International Journal of Stroke |
| Pieri, M.; Lawrence, M.; Grealy, M.; Lowit, A.; Pearl, G. | Heads: Up aphasia, helping ease anxiety and depression following stroke for people with aphasia: A co-creation study to develop an aphasia-friendly mindfulness-based course | 2021 | International Journal of Stroke |
| Pogrebnoy, D.; Cullen, R.; Czerenkowski, J.; Dennett, A.; MacDonald-Wicks, L.; Patterson, A.; Ramage, E.; Zacharia, K.; English, C. | I-Rebound Online. Co-Design of a website prototype to eat well and move more after stroke | 2022 | International Journal of Stroke |
| Porat, T.; Marshall, I. J.; Sadler, E.; Vadillo, M. A.; McKevitt, C.; Wolfe, C. D. A.; Curcin, V. | Collaborative design of a decision aid for stroke survivors with multimorbidity: a qualitative study in the UK engaging key stakeholders | 2019 | BMJ open |
| Poulin, V.; Carbonneau, H.; Provencher, V.; Vincent, C.; Giroux, D.; Rochette, A.; Ouellet, M. C.; Dawson, D.; Nalder, E.; Gagne-Trudel, S. | Promoting leisure participation post-stroke: A web-based program for stroke survivors and their caregivers | 2018 | International Journal of Stroke |
| Power, V.; de Eyto, A.; Hartigan, B.; Ortiz, J.; O'Sullivan, L. W. | Application of a user-centered design approach to the development of XoSoft â€“ A lower body soft exoskeleton | 2019 | Biosystems and Biorobotics |
| Prabhakaran, S.; Kwon, S.; Wymore, E.; Song, S. Y.; Brown, J.; Mason, M.; Kandula, N.; Jones, P.; Eisenstein, A.; Beckstrom, H.; Richards, C. T.; Washington, K. V.; Aggarwal, N. T. | The community engagement for early recognition and immediate action in stroke (ceerias) study: Primary results | 2019 | Stroke |
| Quickenden, J.; Oldham, J.; Marriott, L. | Developing an upper limb pathway for neuro therapy services in Portsmouth-improving staff confidence and patient outcome | 2018 | International Journal of Stroke |
| Radder, B.; Kottink, A. I. R.; Van Der Vaart, N.; Oosting, D.; Buurke, J. H.; Nijenhuis, S. M.; Prange, G. B.; Rietman, J. S. | User-centred input for a wearable soft-robotic glove supporting hand function in daily life | 2015 | 2015 IEEE International Conference on Rehabilitation Robotics |
| Rai, S.; Shiratuddin, M. F.; Carriedo, M.; Harrison, A.; Saleh, K.; Newton, M.; Herne, R.; Blacker, D.; Byrnes, M. | Neuromender::FlexiBrains | 2017 | IEEE 5th International Conference on Serious Games and Applications for Health |
| Rai, T.; Morton, K.; Roman, C.; Doogue, R.; Rice, C.; Williams, M.; Schwartz, C.; Velardo, C.; Tarassenko, L.; Yardley, L.; McManus, R. J.; Hinton, L. | Optimizing a digital intervention for managing blood pressure in stroke patients using a diverse sample: Integrating the person-based approach and patient and public involvement | 2021 | Health expectations : an international journal of public participation in health care and health policy |
| Ramage, E.; Burke, M.; Galloway, M.; Janssen, H.; Lynch, E.; Marsden, D.; Patterson, A.; Pollack, M.; Said, C.; English, C. | Consumer and healthcare worker co-production of a complex intervention for stroke survivors: An integrated knowledge translation approach delivers challenges and potential to change stroke care | 2020 | International Journal of Stroke |
| Ramage, E.; Burke, M.; Galloway, M.; Janssen, H.; Lynch, E.; Marsden, D.; Patterson, A.; Pollack, M.; Said, C.; English, C. | Knowledge translation through co-design: The development of an exercise intervention for stroke survivors | 2019 | International Journal of Stroke |
| Ramage, E. R.; Burke, M.; Galloway, M.; Graham, I. D.; Janssen, H.; Marsden, D. L.; Patterson, A. J.; Pollack, M.; Said, C. M.; Lynch, E. A.; English, C. | Fit for purpose. Co-production of complex behavioural interventions. A practical guide and exemplar of co-producing a telehealth-delivered exercise intervention for people with stroke | 2022 | Health research policy and systems |
| Ríos-Hernández, M.; Jacinto-Villegas, J. M.; Portillo-Rodríguez, O.; Vilchis-González, A. H. | User-centered design and evaluation of an upper limb rehabilitation system with a virtual environment | 2021 | Applied Sciences (Switzerland) |
| Robles, M. C.; Corches, C.; Bradford, M.; Rice, T.; Sukul, D.; Springer, M.; Bailey, S.; Oliver, A.; Skolarus, L. E. | Community and academic partner creation of a community-informed, behavior theory-based music video to encourage stroke preparedness during the COVID-19 pandemic | 2021 | Stroke |
| Robles, M. C.; Springer, M. V.; Corches, C. L.; Burke, J. F.; Lin, C. C.; Oliver, A.; Skolarus, L. E. | Stroke Ready Very Brief Intervention Improves Immediate Postintervention Stroke Preparedness | 2020 | Circulation. Cardiovascular quality and outcomes |
| Rosbergen, I.; Grimley, R.; Tracey, C.; Greveling, N.; Russell, T.; Comans, T.; Walker, K.; Endacott, J.; Brauer, S. | A co-design study to develop a prototype telerehabilitation intervention to increase physical practice after stroke | 2021 | International Journal of Stroke |
| Rose, M.; Carragher, M.; Taylor, N.; Johnson, H.; Torabi, T.; O'Halloran, R. | The Aphasia App: A novel technology-based approach to improving healthcare communication for people with post stroke aphasia and healthcare professionals | 2017 | International Journal of Stroke |
| Ruddell, K.; Shah, C. | Breakfast in bed-24 hour rehab approach on stroke unit: Myth or reality? | 2018 | International Journal of Stroke |
| Sadler, E.; Sarre, S.; Tinker, A.; Bhalla, A.; McKevitt, C. | Developing a novel peer support intervention to promote resilience after stroke | 2017 | Health & social care in the community |
| Said, C.; Ramage, E.; McDonald, C.; Bicknell, E.; Hitch, D.; Fini, N.; Bower, K.; Lynch, E. A.; Vogel, A.; English, K.; McKay, G.; Rebecca Wood, R.; Melathethil, J. J.; English, C. | Development of a shared decision-making tool to support physical rehabilitation via telehealth for stroke survivors with moderate to severe disability | 2021 | International Journal of Stroke |
| Schwertfeger, J. L. | Community coping interventions after stroke study: A pragmatic cohort study using participatory action research | 2021 | Dissertation Abstracts International: Section B: The Sciences and Engineering |
| Semprini, M.; Lencioni, T.; Hinterlang, W.; Vassallo, C.; Scarpetta, S.; Maludrottu, S.; Iandolo, R.; Care, M.; Laffranchi, M.; Chiappalone, M.; Ferrarin, M.; De Michieli, L.; Jonsdottir, J. | User-centered design and development of TWIN-Acta: A novel control suite of the TWIN lower limb exoskeleton for the rehabilitation of persons post-stroke | 2022 | Frontiers in neuroscience |
| Seregni, A.; Tricomi, E.; Tropea, P.; Del Pino, R.; GÃ³mez-Esteban, J. C.; Gabilondo, I.; DÃ­ez-Cirarda, M.; Schlieter, H.; Gand, K.; Corbo, M. | Virtual Coaching for Rehabilitation: The Participatory Design Experience of the vCare Project | 2021 | Frontiers in public health |
| Sharrief, A.; Okpala, M. N.; Vu, K. Y. T.; Savitz, S. I. | The stroke transitions education and prevention clinic: A comprehensive approach to post-stroke care | 2016 | Stroke |
| Sivan, M.; Gallagher, J.; Holt, R.; Weightman, A.; O'Connor, R.; Levesley, M. | Employing the International Classification of Functioning, Disability and Health framework to capture user feedback in the design and testing stage of development of home-based arm rehabilitation technology | 2016 | Assistive technology : the official journal of RESNA |
| Sivan, M.; Gallagher, J.; Holt, R.; Weightman, A.; Levesley, M.; Bhakta, B. | Investigating the International Classification of Functioning, Disability, and Health (ICF) Framework to Capture User Needs in the Concept Stage of Rehabilitation Technology Development | 2014 | Assistive technology : the official journal of RESNA |
| Smith, K.; Brown, G.; Donaldson, R.; Donaldson, K.; Hedberg, A.; Huffman, S.; Lennox, L.; Martin, C.; Mills, J.; Mathany, A. M.; Nicol, C.; Pyle, L.; Walsh, L.; Walsh, T.; Wert, S. L. | Adults with aphasia as change agents: Experiences in advocacy and co-design | 2019 | International Journal of Stroke |
| Song, S. Y.; Araiza, D.; Reyes, C.; Willis, P.; Choi, S.; Kottick, J.; Jimenez, E.; Ma, S.; Trejo, L.; Carrillo, C. A.; et al., | "Worth the walk: " A community-partnered intervention to decrease stroke risk for minority seniors | 2017 | Annals of neurology |
| Song, S.; Araiza, D.; Reyes, C.; Willis, P.; Choi, S.; Kotick, J.; Jimenez, E.; Ma, S.; Trejo, L.; Carrillo, C.; et al., | â€œWorth the walk: â€ a randomized controlled trial of a walking intervention to decrease stroke risk among minority seniors | 2017 | Neurology |
| Springer, M. V.; Islam, N.; Skolarus, L. E. | Adapting a stroke preparedness music video for a high stroke risk population | 2020 | Annals of neurology |
| Springer, M. V.; Corches, C. L.; McCracklin, B.; Scott, Melecia; Robles, M. C.; Skolarus, L. E. | Expanding Stroke Preparedness to Vulnerable Populations: A Music Video for the Deaf Community | 2020 | Journal of the American Heart Association |
| Stewart, C.; Power, E.; McCluskey, A.; Kuys, S. | Development of a participatory, tailored behaviour change intervention to increase active practice during inpatient stroke rehabilitation | 2020 | Disability and rehabilitation |
| Stonecypher, K. | Creating a patient education tool | 2009 | Journal of continuing education in nursing |
| Tanczak, N; Ranzani, R; Meyer, J. T.; Devittori, G; Califfi, A; Dinacci, D; Gassert, R; Lambercy, O; Kanzler, C. M. | A Novel Mixed-Method Approach to Identify Needs and Requirements for Upper Limb Assistive Technology for Persons after Stroke | 2022 | IEEE ... International Conference on Rehabilitation Robotics : [proceedings] |
| Teed, M.; Arthur, G.; Takacs, J.; Jacques, S.; Tardif, A.; Young, C.; Hamilton-Page, M. | Evidence-based development of online peer support for caregivers: Co-creating success | 2019 | International Journal of Stroke |
| Teed, M.; Ratz, J.; Arthur, G.; Hamilton-Page, M. | Women and online engagement: partnering to improve health | 2021 | Canadian Journal of Cardiology |
| Teeling, S. P.; Coetzee, H.; Phillips, M.; McKiernan, M., Ní ShÉ, É., Igoe, A. | Reducing risk of development or exacerbation of nutritional deficits by optimizing patient access to mealtime assistance | 2019 | International journal for quality in health care : journal of the International Society for Quality in Health Care |
| Termoz, A.; Delvallee, M.; Damiolini, E.; Marchal, M.; Preau, M.; Huchon, L.; Mazza, S.; Habchi, O.; Bravant, E.; Derex, L.; Nighoghossian, N.; Cakmak, S.; Rabilloud, M.; Denis, A.; Schott, A. M.; Haesebaert J. | Co-design and evaluation of a patient-centred transition programme for stroke patients, combining case management and access to an internet information platform: study protocol for a randomized controlled trial - NAVISTROKE | 2022 | BMC health services research |
| Tetteroo, D. | Designing end-user adaptable interactive rehabilitation technology | 2019 | Technology and disability |
| Tetteroo, D.; Willems, L.; Markopoulos, P. | Patient feedback design for stroke rehabilitation technology | 2015 | Biomedical Engineering Systems and Technologies |
| Thayabaranathan, T.; Immink, M. A.; Hillier, S.; Stolwyk, R.; Andrew, N. E.; Stevens, P.; Kilkenny, M. F.; Gee, E.; Carey, L.; Brodtmann, A.; Bernhardt, J.; Thrift, A. G.; Cadilhac, D. A. | Co-designing a new yoga-based mindfulness intervention for survivors of stroke: A formative evaluation | 2022 | Neurology international |
| Thornton, M.; Harris, J.; Breithaupt, K.; Dyks, T.; Finestone, H.; MacKay-Lyons, M. | Development of a digital learning program for physiotherapists to enhance clinical implementation of aerobic exercise in stroke rehabilitation | 2021 | Archives of physiotherapy |
| Tsoupikova, D.; Stoykov, N.; Vick, R.; Li, Y.; Kamper, D.; Listenberger, M. | Use of virtual reality to promote hand therapy post-stroke | 2013 | Studies in Health Technology and Informatics |
| Turton, A.; Manns, S.; Hampshire, L.; O'Connor, R.; Helps, T; Rossiter, J | Focus groups for co-design of robotic trousers for improving mobility in older people...RCOT (Royal College of Occupational Therapist) Annual Conference 2017 | 2017 | British Journal of Occupational Therapy |
| Vidal, M.; Robson, L.; McIntosh, C. | Ending well and moving on: Creating a patient-centred discharge summary with stroke survivor input | 2016 | International Journal of Stroke |
| Von Koch, L.; Laska, A. C.; Lindblom, S.; Flink, M.; Tistad, M.; Ytterberg, C. | Co-designed person-centred care transitions for people with stroke - a feasibility study | 2022 | European stroke journal |
| Walsh, R. J.; Depaul, O.; Baum, C.; Nicol, G. E.; Keenoy, K.; Klein, M.; Wong, A. | Adapting and Implementing a Mobile Self-Management Program for Daily Life Participation in Individuals With Stroke...American Occupational Therapy Association (AOTA) INSPIRE 2021 (Virtual), April 6-30, 2021 | 2021 | American Journal of Occupational Therapy |
| Wang, H.; Ghazi, M.; Chandrashekhar, R.; Rippetoe, J.; Duginski, G. A.; Lepak, L. V.; Milhan, L. R.; James, S. A. | User Participatory Design of a Wearable Focal Vibration Device for Home-Based Stroke Rehabilitation | 2022 | Sensors (Basel, Switzerland) |
| Warland, A.; Kilbride, C.; Tsekleves, E.; Skordoulis, D.; Paraskevopoulos, I. | ReWiiRe (Research in Wii Rehabilitation): User involvement in the development of a personalised rehabilitation system for arm re-education after stroke | 2012 | International Journal of Stroke |
| Wentink, M.; van Bodegom-Vos, L.; Brouns, B.; Arwert, H.; Houdijk, S.; Kewalbansing, P.; Boyce, L.; Vliet Vlieland, T.; de Kloet, A.; Meesters, J. | How to improve eRehabilitation programs in stroke care? A focus group study to identify requirements of end-users | 2019 | BMC medical informatics and decision making |
| Wilkinson, A.; Rennick Egglestone, S.; Mawson, S.; Burridge, J.; Fitzpatrick, G. | Motivating mobility-clinical evaluation of two case studies developing new technologies to motivate rehabilitation post-stroke | 2011 | Physiotherapy (United Kingdom) |
| Williamson, T.; Ryan, J.; Smith, C.; Cooper, G.; Howard, D.; Kenney, L. P. J.; Matthews, M.; Barker, A. T.; Good, T.; Heller, B. W. | Public involvement in the development of a new functional electrical stimulation system | 2012 | Neuromodulation |
| Wilson, S., Roper, A., Marshall, J., Galliers, J., Devane, N., Booth, T., Woolf, C. | Codesign for people with aphasia through tangible  design languages | 2015 | Co-design - International Journal of Co-creation in Design and the Arts |
| Wong, A.; DePaul, O.; Hirsch, S.; Walsh, R.; Knarr, S.; Knarr, M.; Perdue, R.; Baum, C. M.,; Ginger, N. | Harnessing an Implementation Science Framework and Measures to Develop a Technology-Augmented Self-Management Intervention for Stroke Rehabilitation | 2022 | Archives of Physical Medicine & Rehabilitation |
| Wray, F.; Clarke, D.; Cruice, M.; Forster, A. | Development of a self-management intervention for stroke survivors with aphasia using co-production and behaviour change theory: An outline of methods and processes | 2021 | PloS one |
| Wu, N.; Gong, E.; Wang, B.; Gu, W.; Ding, N.; Zhang, Z.; Chen, M.; Yan, L. L.; Oldenburg, B.; Xu, L. | A Smart and Multifaceted Mobile Health System for Delivering Evidence-Based Secondary Prevention of Stroke in Rural China: Design, Development, and Feasibility Study | 2019 | JMIR mHealth and uHealth |
| Yang, C.; Mantha, A.; Savage, J.; Martineau, I.; Pettem, H.; Powell, J.; McDonnell, L. | Enhancing stroke outpatient services at Bruyere Continuing Care: A Design Thinking approach | 2019 | International Journal of Stroke |
| Young, C.; Hamilton-Page, M.; Jacques, S.; Bromley, R. A.; Wong, G.; McElhinney, P.; Teed, M.; Arthur, G. | Evidence based development of online peer support: Co-creating success | 2018 | International Journal of Stroke |
| Young, R.; Smith, C.; Sage, K.; Broom, D. | Application of the nominal group technique to inform a co-design project on power assisted exercise equipment for people with stroke | 2021 | Physiotherapy (United Kingdom) |
| Young, R.; Sage, K.; Broom, D.; Broomfield, K.; Church, G.; Smith, C. | Using nominal group technique to advance power assisted exercise equipment for people with stroke | 2021 | Research involvement and engagement |
| Zacharia, K.; Patterson, A. J.; English, C.; Ramage, E.; Galloway, M.; Burke, M.; Gray, R.; MacDonald-Wicks, L. | i-Rebound after Stroke-Eat for Health: Mediterranean Dietary Intervention Co-Design Using an Integrated Knowledge Translation Approach and the TIDieR Checklist | 2021 | Nutrients |
| Zimmer, L.; Kirenko, W. | An electronic clinical toolkit to support acute stroke management within a rural community setting | 2011 | Stroke |

Supporting information 2: List of all data sources that used co-design for stroke intervention development, including data sources that aligned with co-design or associated methodology with and without explicitly using the term co-design
